# Neuroserpin, a crucial regulator for axogenesis, synaptic modelling and cell–cell interactions in the pathophysiology of neurological disease

**DOI:** 10.1007/s00018-022-04185-6

**Published:** 2022-03-04

**Authors:** Angela Godinez, Rashi Rajput, Nitin Chitranshi, Veer Gupta, Devaraj Basavarajappa, Samridhi Sharma, Yuyi You, Kanishka Pushpitha, Kunal Dhiman, Mehdi Mirzaei, Stuart Graham, Vivek Gupta

**Affiliations:** 1 Faculty of Medicine, Health and Human Sciences, Macquarie University, F10A, 2 Technology Place, North Ryde, NSW 2109 Australia; 2 School of Medicine, Deakin University, Melbourne, VIC Australia; 3 Save Sight Institute, University of Sydney, Sydney, NSW Australia

**Keywords:** Neuroserpin, Plasminogen, Tissue plasminogen activator, Glaucoma, Alzheimer’s disease, Stroke, Serpin, Retina

## Abstract

Neuroserpin is an axonally secreted serpin that is involved in regulating plasminogen and its enzyme activators, such as tissue plasminogen activator (tPA). The protein has been increasingly shown to play key roles in neuronal development, plasticity, maturation and synaptic refinement. The proteinase inhibitor may function both independently and through tPA-dependent mechanisms. Herein, we discuss the recent evidence regarding the role of neuroserpin in healthy and diseased conditions and highlight the participation of the serpin in various cellular signalling pathways. Several polymorphisms and mutations have also been identified in the protein that may affect the serpin conformation, leading to polymer formation and its intracellular accumulation. The current understanding of the involvement of neuroserpin in Alzheimer’s disease, cancer, glaucoma, stroke, neuropsychiatric disorders and familial encephalopathy with neuroserpin inclusion bodies (FENIB) is presented. To truly understand the detrimental consequences of neuroserpin dysfunction and the effective therapeutic targeting of this molecule in pathological conditions, a cross-disciplinary understanding of neuroserpin alterations and its cellular signaling networks is essential.

## Introduction

Neuroserpin or axonin-2 is a glycosylated serine proteinase inhibitor (serpin) that was initially identified as a protein secreted from cultured dorsal root ganglia of chicken embryo [145]. Subsequent in situ hybridisation and northern blot analysis showed this protein to be predominantly localised within the neuronal populations of the central and peripheral nervous system. However, the protein has also been reported to be expressed in pancreas, kidneys, skeletal muscle, heart tissue, and immune cell populations within the blood [98, 117].

Primary structural evaluation of neuroserpin has revealed its molecular mass to be approximately 44 kDa, which is increased further by N-linked glycosylation. The protein is also composed of 410 amino acids, which is reduced to 394 amino acids following the cleavage of signal peptides [139, 145]. Purified human neuroserpin exhibits a molecular weight of 55 kDa, owing to glycosylation at multiple sites [117, 139, 157]. Neuroserpin is encoded by the *SERPINI1* gene, and mapped to q26 region of chromosome 3 with a total of nine exons [139].

Serpin proteins have been implicated in regulating multiple physiological processes such as coagulation, fibrinolysis, complement system activation and the modulation of serine protease activity in a cell- and tissue-specific manner [115, 141, 144]. Studies have established that neuroserpin as a member of the serpin family, shares a high degree of homology with the archetypal serpin, alpha-1 antitrypsin [30]. Neuroserpin was initially predicted to be heparin-independent and a functional inhibitor of trypsin-like proteases within the nervous system [117], with subsequent studies demonstrating its inhibition of amidolytic activity of tissue plasminogen activator (tPA) and plasmin [87, 116]. Neuroserpin has since been shown to play key roles in mediating neurite outgrowth, axonal development and maintaining normal synaptic plasticity through its inhibitory effects on tPA in the central nervous system (CNS), but also through mechanisms that are partially independent of tPA [64, 127, 139, 171]. Structurally, neuroserpin possesses a conserved ‘serpin fold’, that is composed of three β-sheets and nine α-helices within the main body of the protein [48, 129, 141]. The serpin also contains an exposed and structurally flexible reactive centre loop (RCL), which utilises a mouse-trap mechanism of suicide substrate inhibition [48, 115], that allows the protein to present itself as a pseudo substrate to the target serine proteases, such as plasmin and tPA [115]. The interacting protease engages with the scissile P1–P1’ peptide bond of the serpin in the RCL and cleaves it through the formation of an intermediate covalent Michaelis complex. This cleavage induces a conformational change in the serpin structural fold that in turn distorts the active site of the proteolytic enzyme, rendering it catalytically inactive [48, 71].

**Fig. 1:**
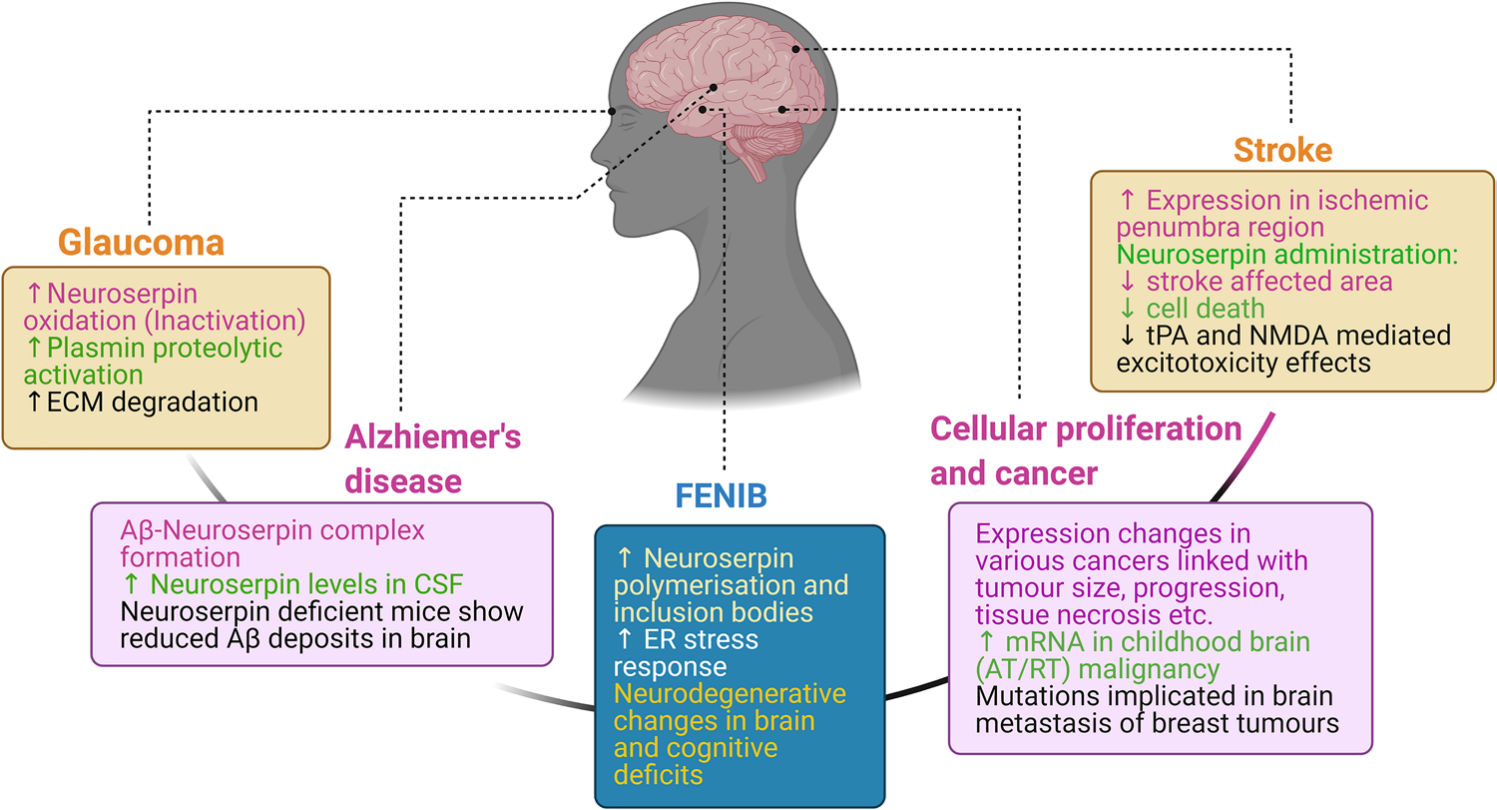
The role of neuroserpin in various diseases of the central nervous system. A flow diagram showing the involvement of neuroserpin in various disorders affecting the central nervous system. AT/RT, Atypical teratoid/rhabdoid tumours

Alterations in neuroserpin activity and its expression have been reported, with potentially deleterious effects in various neuropathological disease conditions. The protein deficiency for instance, has been implicated in inducing exacerbated neuronal cell death and increased cerebral infarct size following focal cerebral ischemia in mouse models of stroke [47]. On the other hand, treatment with neuroserpin following kainic acid-induced seizures has been shown to significantly delay the progression of seizure activity [173], and exerts a neuroprotective effect against *N*-methyl-d-aspartate (NMDA) induced excitotoxicity, both in vivo and in vitro [89]. Exogenous neuroserpin administration has also been associated with the reduction of cerebral infarct volumes, as well as increased neuronal survival in post-embolic and ischemic-induced conditions [25, 131, 175, 179]. Changes in neuroserpin expression and activity have also been associated with cancer metastasis, Alzheimer’s disease (AD) and primary open angle glaucoma (POAG) pathologies (Fig. 1) [39, 55, 65, 90, 153]. Furthermore, neuroserpin genetic variations have been shown to be associated with phenotypic clinical manifestations including progressive myoclonus epilepsy (neuroserpinosis), in familial encephalopathy with neuroserpin inclusion bodies (FENIB) (Fig. 1) [30, 132]. Emerging evidence also points towards a possible role for neuroserpin in modulating neurovascular permeability, inducing neuroprotective effects, promoting axonal regeneration and effects independent of its canonical interactions with tPA [100, 121, 131]. By elucidating the cellular pathways that may influence or alter the role of neuroserpin as a protease inhibitor or underlie the impact of neuroserpin genetic variants, it may be possible to improve our knowledge of various neurological disease mechanisms with wider applicability to identify novel therapeutic approaches. This review focuses on neuroserpin actions both dependent and independent of tPA and plasminogen in various neuropathological disorders and discusses recent research outcomes, which could help us develop mechanism-based protective strategies.

## Neuroserpin involvement in axonal growth and synaptic plasticity

Whilst in vitro studies demonstrate the complex formation and inhibition of tPA, it is unclear which neuroserpin functions may be dependent or independent of tPA in vivo [64, 116]. Neuroserpin has been demonstrated to interact with and inhibit the proteolytic activity of urokinase plasminogen activator (uPA), plasmin and trypsin-like serine proteinases in vitro [64, 116]. The tPA/uPA system is intricately associated with neuronal development, promoting synaptic connectivity, plasticity and axonal refinement [159]. Chiefly, the plasmin/plasminogen activator network is involved in the proteolytic degradation of the extracellular matrix (ECM), as a driving force for remodulation, cell migration and growth cone motility [109]. Hence, neuroserpin is thought to fine-tune the proteolytic activity of these enzymes and mediate neurogenesis and synapse formation [67, 167]. In particular, the co-expression of neuroserpin with neuronal markers, such as Tuj1 and calbindin, suggested its involvement during the early stages of neurogenesis in the hippocampi of adult rats [167]. Neuroserpin deficiency during early neurodevelopmental phases is also associated with deficits in neurogenesis via the premature differentiation of hippocampal neurons. Premature termination of the neuronal precursor proliferative phase was found to be concomitant to changes in the ECM composition and dendritic spine morphology, to a more mature phenotype [67]. These observations have been corroborated in cell culture experiments where neuroserpin was shown to induce neurite outgrowth in AtT20 cells [69]. However, it is still unknown whether the inhibitory function of neuroserpin is necessary for the protein to mediate neurogenesis and synaptic formation, particularly since neuroserpin was also demonstrated to promote cell–cell interactions in cultured pheochromocytoma PC12 cells in a tPA-independent manner [90].

The spatio-temporal distribution of neuroserpin mRNA and protein in the CNS was detailed in early studies by Krueger et al. [87]. Neuroserpin was observed to be abundantly expressed during late-stage post-mitotic development, following the cessation of axonal pathfinding and during synapse formation processes. A weak detection of neuroserpin mRNA was evident in the neuronal precursors migrating from ventricular zones, and into the cortical plate. The post-mitotic neurons of the neocortex, which had settled in the cortical plate strongly expressed neuroserpin as they began extending axons that would eventually develop synaptic networks. Neuroserpin expression has been shown to reach its highest levels perinatally and regress after the development of the cortical plate, following the first week of life in mice [87]. Increased neuroserpin expression is also reported within the neuromuscular junctions of motor neurons in mice during early postnatal development. In adults, the overall expression is weaker, but stronger neuroserpin expression is visible in areas of the neocortex, amygdala, olfactory bulb and hippocampus that undergo active synaptic plasticity [82, 87].

The enhanced expression of neuroserpin has been suggested to elicit neural changes associated with improved cognitive function, namely the higher density of dendritic protrusions, as well as increased length and alterations of dendritic spine shape in hippocampal neurons [13]. This indicates positive effects of neuroserpin on synaptic plasticity that correlate with improved memory, learning, social behaviour, and cognition [64, 101, 127]. Accordingly, the deficiency in neuroserpin expression was demonstrated to affect learning and memory retention capacity in mice [127]. Furthermore, mice lacking or overexpressing neuroserpin were shown to manifest behavioural phenotypic changes including deficits in exploratory behaviour, neophobia and the augmentation of anxiety-like symptoms [101].

## The role of neuroserpin in neuropsychiatric conditions

As previously described, neuroserpin is abundantly expressed in the cerebral hippocampus, neocortex, olfactory bulb and amygdala regions, that are intricately associated with memory processing and learning [87, 127]. Neuroserpin is also shown to be significantly expressed in the noradrenergic neurons of the *locus coeruleus*, an area that has been implicated to play a role in vigilance, fear and the processing of sensory stimuli, alluding to a role for neuroserpin in various neuropsychiatric conditions [15, 87].

Early studies demonstrated that neuroserpin is involved in regulating mood and emotional health, with implications in anxiety-like behaviour. Transgenic mice that either overexpressed or were deficient in neuroserpin indeed displayed atypical neophobic behaviour with reduced locomotor activity, decreased exploration of novel environments, anxiety-like responses to O-maze situations and the avoidance of novel objects. In particular, mice that overexpressed neuroserpin were averse to bright light, and spent a significant amount of their time hidden in dark compartments in the light–dark box tests [101]. A similar anxiety-like phenotype was reported in neuroserpin-deficient zebrafish that exhibited a preference to stay in outer zones of the test arena, in both continuous light and light-to-dark transition conditions. Further, RNA sequencing analysis revealed differentially expressed pathways in neuroserpin-deficient zebrafish larvae, including the downregulation of G protein signalling 2 (Rgs2) [60]. Rgs2 has previously been implicated not only in AD-related cognitive deficits [58], but it also influences anxiety-like symptoms in mice [166], as well as generalised anxiety and panic disorder in humans [85, 93]. The gene is also associated with an array of intermediate phenotypes, such as childhood temperament, adult personality, introversion, agoraphobia, and alterations to functions of the amygdala and insular cortex in humans [93, 142].

Recently, neuroserpin-deficient mice have been described to exhibit sociability deficits in behavioural assays, specifically in social investigation when introduced to unfamiliar mice, concomitant to reduced long term potentiation. As such, this study also investigated neuroserpin protein levels in post-mortem brain samples of patients with idiopathic autism and schizophrenia; however, no significant changes were observed when compared to age- or gender-matched controls [127]. In contrast, neuroserpin gene upregulation in the brain tissue of chronic schizophrenic subjects was previously observed, using RNA microarray analysis in genome-wide association studies [59]. Earlier studies have indicated neurodevelopmental and synaptic pathology in schizophrenia [162], and this adds weight to the significance of neuroserpin in the disorder, particularly due to its inherent role in synaptic plasticity during developmental and adult stages of life [87]. Neuroserpin was also notably upregulated in the induced pluripotent stem cells (iPSCs) generated from schizophrenic patients, that were analysed via deep RNA sequencing [163].

A robust neuroserpin expression within the serotonergic neurons of the *raphe nuclei* has been observed and this is significant, considering the role of the dorsal *raphe nuclei* both as a canonical transmitter of serotonin, and in its central involvement in neuroplasticity [87]. Serotonergic neurons are directly involved in regulating mood and indicated in conditions such as major depressive disorder [83, 105]. Indeed, various drugs, such as selective serotonin re-uptake inhibitors (SSRIs), that manipulate the serotonergic network are the first line of anti-depressant treatment in these conditions [46]. Recent investigations found that neuroserpin expression is diminished in rat models of depression and neuro-inflammation. These reports were further substantiated by decreased neuroserpin mRNA expression in the peripheral blood mononuclear cells of patients with first-episode depression. Further, attenuated neuroserpin mRNA in these patients was negatively correlated with Beck depression inventory scores [61]. Meanwhile, neuroserpin was observed to be upregulated in a rat serotonergic cell line derived from the *raphe nucleus* (RN46A) in response to valproic acid treatment, a mood-stabilising drug that is commonly prescribed for bipolar disorder [4]. Microarray analysis of anti-depressant related genes also revealed enriched expression of neuroserpin in the adult rat hippocampus, specifically in the dentate gyrus sub-granular zone. This area is critical in hippocampal neurogenesis for adults, and extremely receptive to environmental and pharmacological stimulation [167]. Indeed, the inhibition of neurogenesis in this region has been demonstrated to block the therapeutic action of anti-depressant drugs [136]. Collectively, these studies reveal the involvement and significance of neuroserpin-mediated changes in the aetiology of mood disorders. Future investigations into the genetic and cellular signalling pathways will help to elucidate the molecular mechanisms underlying the role of neuroserpin in these debilitating neuropsychiatric conditions.

## Neuroserpin crosstalk with cellular signalling networks

**Fig. 2:**
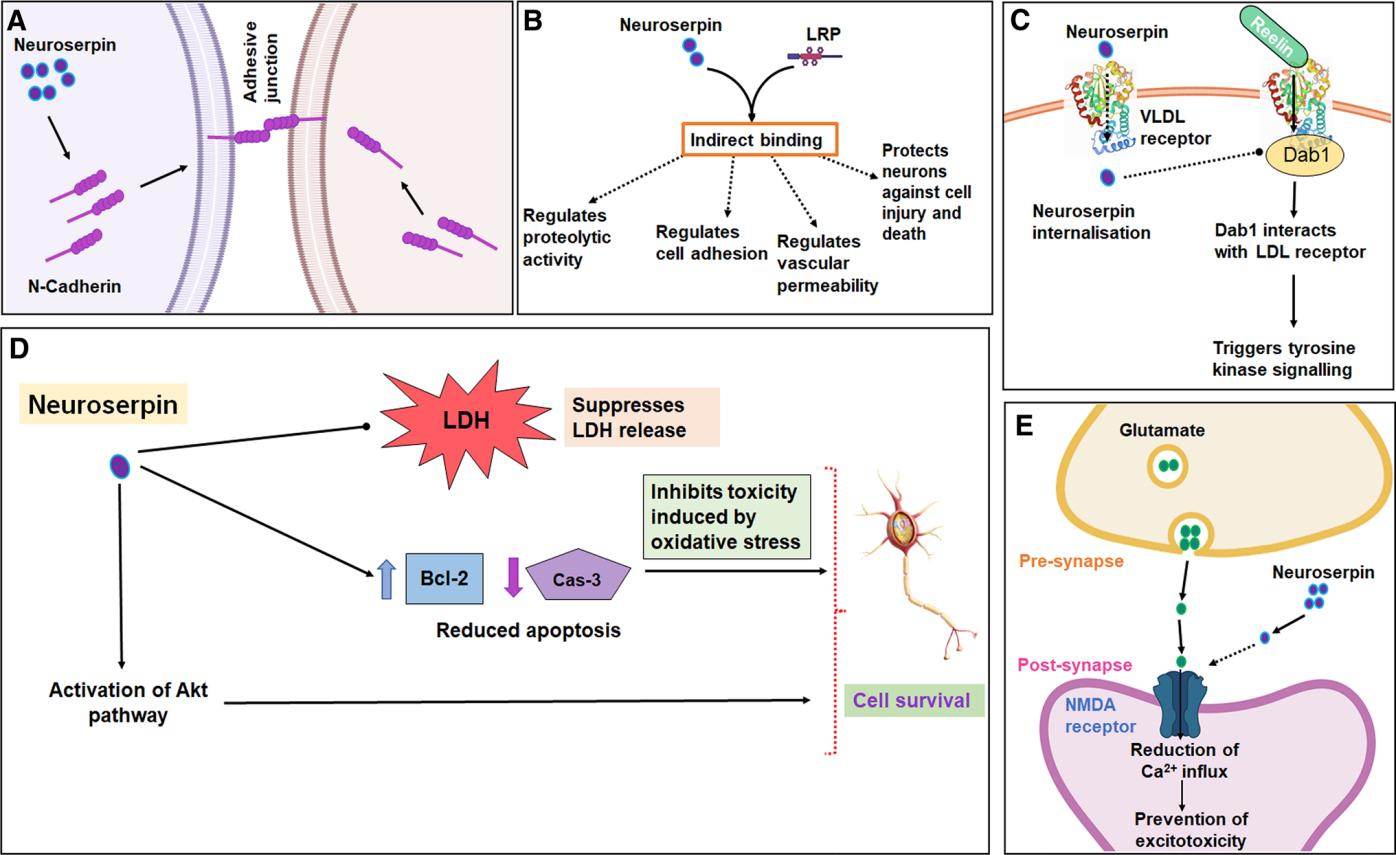
Neuroserpin crosstalk with cellular signalling pathways. **A** Neuroserpin is involved in cell adhesion via the regulation of N-cadherin expression. **B** Neuroserpin interacts with LRP (lipoprotein receptor-related protein) and may regulate localised proteolytic activity, cell adhesion, vascular permeability and neuronal protection against cell injury and death (dotted lines). **C** VLDL (very-low-density-lipoprotein) receptors have been shown to endocytose neuroserpin. It is hypothesised that neuroserpin may mediate reelin signalling leading to the activation of disabled-1 protein (Dab1). Dab1 interacts with the LDL (low-density lipoprotein) receptor and promotes intracellular tyrosine kinase signalling in brain and retina. **D** In stressed conditions, neuroserpin plays an important role in the activation of the Akt signalling, suppression of cell death (reduced lactose dehydrogenase release), prevention of apoptotic pathways induced by oxidative stress in neurons, and the activation of anti-apoptotic proteins. **E** Neuroserpin has been suggested to interact with NMDA (*N*-methyl-d-aspartate) receptors leading to reduction in Ca^2+^ influx and suppress excitotoxicity in neurons. Dotted lines in the figure indicate hypothesised functions, that have not been demonstrated experimentally

Several downstream cellular signalling events linked with neuroserpin have been identified in the brain and in retinal tissues in recent years. These effects are mediated either through the proteinase inhibitory effects of neuroserpin on plasminogen activators, or its non-enzyme inhibitory roles [55, 117, 165]. Neuroserpin mainly exerts neuroprotective effects through its canonical inhibitory actions on plasminogen activators such as tPA [25, 175]. Recent literature suggests that the neuroprotective effects of neuroserpin might also be attributed to its direct inhibitory actions on plasmin and its ability to moderate plasmin-mediated excitotoxicity, independent from tPA [165]. Plasmin inhibitory activity of neuroserpin was reported by us in the retina, which might play a neuroprotective role for retinal ganglion cells in glaucoma [55]. As an inhibitor of tPA and plasmin, the serpin may contribute to the preservation of neurovascular unit integrity through its modulatory effects on neuronal nitric oxide (NO) synthase and proteolytic activation of platelet-derived growth factor signalling [42, 118, 120, 147]. With respect to non-proteinase inhibitory roles, the serpin is implicated in regulating the extent of cell–cell adhesion in vitro and this role appears to be independent of the inhibition of plasminogen activators, leading to the proposition that these effects might be mediated through different cell membrane receptors [90]. Furthermore, the cytoskeletal-adhering protein, N-cadherin is increased in cells overexpressing neuroserpin, suggesting a role for neuroserpin in regulating cell adhesion-associated signalling pathways. Increased N-cadherin levels were particularly localised to cell membrane compartments in contact with other cells, consistent with its roles in mediating cell adhesion (Fig. 2a). Interestingly, the effect on N-cadherin and the regulation of cell adhesion was also evident with mutant neuroserpin species, which were devoid of innate proteinase inhibitory activity; further supporting the hypothesis that neuroserpin is involved in regulating critical cellular functions through pathways beyond tPA and plasmin engagement [90]. N-cadherin plays important roles in synapse formation and forming Ca^2+^-dependent interactions with actin and catenin cytoskeletal networks [17]. Neuroserpin is enriched in presynaptic terminals and its regulation of cadherins implicates that the serpin might play a role in the formation and preservation of synaptic connections. These effects could possibly be mediated through the Rho family of GTPases, that are regulated through N-cadherin signalling [17]. Taken together, these interactions implicate the role of neuroserpin in preserving synaptic networks, through cell adhesion and intracellular signalling pathways, which have yet to be fully understood [17, 90].

Of interest, the low-density lipoprotein receptor-related protein (LRP) has been observed to modulate neuroserpin levels by mediating the internalisation of both neuroserpin and neuroserpin–tPA complexes across the cell membrane in neuronal cultures and fibroblasts. The regulation of neuroserpin internalisation by LRP is postulated to regulate localised proteolytic activity in synaptic regions [102]. LRP regulates many cellular signalling pathways [2, 66, 70], and it is hypothesised that neuroserpin may interact with LRP to maintain a fine equilibrium of its cellular actions (Fig. 2b) [100]. Both neuroserpin and LRP have been identified to be localised in synaptic regions and their co-localisation suggests a potential role in collectively modulating proteolytic turnover. Of note, although LRP has been shown to mediate neuroserpin endocytosis, the protein was observed to not directly bind to LRP, suggesting that there might be another interacting partner required for neuroserpin internalisation. Further studies in cells lacking plasminogen activators established that this additional interacting partner is not tPA, suggesting that there might be another protein or co-factor that is involved in facilitating neuroserpin binding to LRP in vivo and its subsequent internalisation [102]. These additional interacting partners can be investigated by immunoprecipitation of neuroserpin and LRP using specific antibodies followed by proteomics analysis. As such, the identification of one or more of these factors will help dissect molecular mechanisms underlying the non-protease inhibitory actions of neuroserpin. It is important to highlight that generally, cells uptake serpins as a complex with cognate proteases for clearance and therefore LRP mediated endocytosis of neuroserpin seems to be an exception [146]. Future studies should investigate whether neuroserpin undergoes reversible conformational changes or a certain degree of oligomerisation in cultured conditions, which might alter its affinity with LRP or with other interacting partners as possible explanations.

Another related receptor, very-low-density lipoprotein receptor (VLDLR) that is expressed in neurons, has also been identified to facilitate neuroserpin uptake in cells. This receptor interestingly, mediates endocytosis of neuroserpin alone and not in its complex form with tPA, suggestive of its different affinity towards the molecule compared to LRP-1 [102]. VLDLR participates in reelin signalling in the brain that induces activation of the disabled-1 protein (Dab1). Dab1 protein in turn interacts with LDL receptors and stimulates several tyrosine kinase signalling networks [45]. Thus, it is postulated that neuroserpin may indirectly participate in and control signalling downstream of VLDLR, reelin and Dab1 by regulating their interactions [37]. These interactions will likely lead to activation or inhibition of specific biochemical cascades in the brain and retina in a tissue- and cell-specific manner (Fig. 2c). Neuroserpin has also been observed to mediate several cellular effects through its crosstalk with neurotrophins. For example, nerve growth factor (NGF)-induced neurite extension was increased in response to reduced neuroserpin expression in vitro using PC12 cell lines [121]. Later investigations established the activity-dependent release of precursor NGF in vivo. Here, neuroserpin was shown to regulate proteolytic processing of the neurotrophin within the cortical extravascular space of rat cortices and enabled its conversion from precursor to mature NGF [16]. Neuroserpin has also been shown to protect hippocampal neurons against oxidative stress, even in the absence of tropomyosin receptor kinase (Trk) receptors, suggesting that the serpins role in oxidative stress is not mediated through these receptors [22]. In contrast, TrkA receptors could hypothetically be indirectly stimulated through neuroserpin and neuroserpin:tPA complex interactions with LRP [22, 91]. Similar activation of protein kinase B (Akt) and Trk receptors by α2 macroglobulin and tPA binding with LRP has been reported previously [140]. Given that Trk receptor activation is closely involved in regulating neuronal survival and the inhibition of apoptosis, these changes acting in tandem can promote cellular survival (Fig. 2d) [55, 56]. This is consistent with the reported neuroprotective effects of neuroserpin, albeit this hypothesis has yet to be demonstrated experimentally and warrants further investigations. Moreover, neuroserpin indirectly modulates the phosphoinositide 3-kinase/protein kinase B (PI3K/Akt) pathway which promotes neuroprotection. However, the mechanisms underlying this activation are unclear. Recent studies have detailed that exogenous neuroserpin treatment of hippocampal neurons in culture, imparted protection against oxidative stress induced damage through its effects on Akt signalling, whereas PI3K inhibitor treatment suppressed the neuroprotective effects of neuroserpin [22]. The serpin treatment was also shown to enhance neuronal survival and suppress apoptosis and LDH release, as well as modulate the expression of apoptotic pathway-associated B cell lymphoma 2 (Bcl-2) and caspase-3 proteins in mitochondria. As such, neuroserpin treatment was effective in enhancing the expression of anti-apoptotic protein Bcl-2, which was initially suppressed upon exposure to oxidative stress in hippocampal cultures. Rather significantly, the exacerbation of caspase-3 levels in these cells in response to oxidative stress was reduced by neuroserpin. Together, these observations strengthen the hypothesis that the protective effects of neuroserpin in neuronal cells might be mediated through its downstream effects on Bcl-2 and caspase-3 signalling [22].

Likewise, neuroserpin promoted the survival of cells that were exposed to oxygen–glucose deprivation [158]. Here, while p65 and p-IKKBα/β expression was elevated upon withdrawal of oxygen and glucose, the stress response was diminished consequent to neuroserpin treatment. These neuroprotective effects are attributed to downstream alterations in tumour necrosis factor-alpha (TNFα), NO and NFκB levels and due to the inhibition of the mitogen-activated protein kinase (MAPK) signalling pathways [158]. In particular, some of the neuroserpin modulatory effects on astrocytic morphology and neuroprotection were lost upon NFκB inhibition, signifying that the downstream protective effects of the serpin might in part be induced via NF-κB signalling [158].

The neuroserpin protein also possesses approximately 20 methionine (Met) residues, which could possibly play a role in mediating its antioxidant potential and resultant neuroprotective effects [108]. We observed that neuroserpin undergoes increased oxidation at the active site Met residue in glaucoma conditions in the retinas of both human and animal model tissues [55]. This observation was further validated in superoxide dismutase (SOD) mutant mice, where neuroserpin deactivation was associated with increased oxidative stress [55]. As such, further understanding of the cellular signalling networks regulating neuroserpin actions might offer new opportunities to target this molecule in stroke and chronic neurodegenerative conditions of the brain and retina, such as Alzheimer’s disease (AD), Familial encephalopathy with neuroserpin inclusion bodies (FENIB) and glaucoma.

## Neuroserpin implications in Alzheimer’s disease pathology

Alzheimer’s disease (AD) is one of the most prevalent forms of dementia, characterised by progressive mental and cognitive deficits, particularly in ageing populations [81]. The progression of AD pathogenesis is believed to be driven at least in part by the accumulation of toxic extracellular amyloid-beta (Aβ) protein in the brain [41]. Aβ fibrillary structures and plaques have been shown to contain the neuroserpin protein, which was demonstrated to form a 1:1 binary complex with N-terminals of Aβ peptides [84].

Chiou et al. [24] investigated neuroserpin-Aβ interactions using the single molecule fluorescence method and demonstrated that the attachment of Aβ into the β-sheets of neuroserpin accelerates its interaction, with the formation of a polymerogenic neuroserpin monomer, followed by Aβ displacement [24]. This suggests that Aβ could act as a facilitator for neuroserpin polymerisation, further pointing towards an important, yet unelucidated role for neuroserpin in neurogenerative disease [24]. However, there is conflicting evidence to suggest whether neuroserpin activity within AD is neuroprotective, or conversely plays a role in disease progression. Initial studies have shown an increased level of neuroserpin in cerebrospinal fluid (CSF), as well as brain tissue of AD patients, when compared to controls [39, 114]. This was in contrast to the normal expression of other serpin family members’ plasminogen activator inhibitor-1 (PAI-1) and protease nexin-1 (PN-1) [39]. Further, neuroserpin levels were positively correlated with tau biomarkers in the CSF [114]. Later studies also corroborated these findings and reported a significant upregulation of neuroserpin, alongside plasminogen in the CSF of patients with mild cognitive impairment (MCI), in comparison to subjective cognitive impairment (SCI) subjects. However, these studies also showed that neuroserpin and plasminogen expression in AD patients was not significantly different to those with SCI [62]. In human post-mortem studies, neuroserpin mRNA was reported as significantly decreased in the frontal and temporal cortices of AD patients [6]. The neuroserpin protein levels were also shown to decline as the Braak staging increased, suggesting that neuroserpin levels may decrease in the advanced stages of AD, following extensive neuronal loss. On the other hand, studies in AD transgenic mice with neuroserpin gene ablation have demonstrated rapid clearance of Aβ_40_ and Aβ_42_ levels, as well as a decrease in Aβ plaque burden, and more active tPA interaction with the plaques. These animals also manifested an overall improvement in behavioural testing performance compared to AD mice with neuroserpin, suggesting that increased amyloid clearance in neuroserpin ablated AD mice may be due to more efficient action of tPA or plasmin on Aβ oligomers [38]. Collectively these studies highlight a complex and not yet fully understood role of neuroserpin in AD pathogenesis. The specific increase of neuroserpin levels within CSF in MCI, however, indicates its potential novel applications as a biomarker for the early detection of AD pathology [62, 114].

**Fig. 3:**
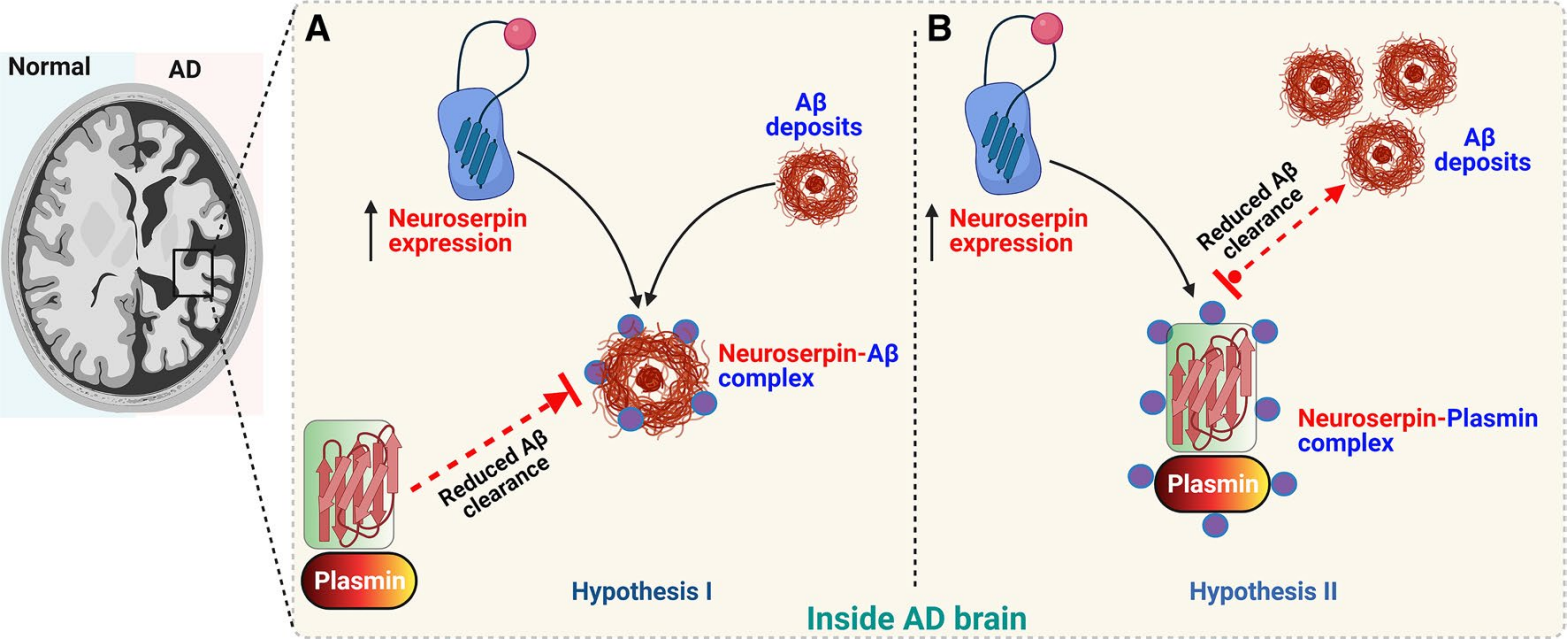
Neuroserpin involvement in regulating amyloid β pathology. Schematic figure showing **A** neuroserpin binding to amyloid β fibrils leading to neuroserpin-Aβ complex formation. This neuroserpin-Aβ complex is resistant to plasmin proteolytic action and reduces the clearance of amyloid β deposits. **B** Increased neuroserpin binds to plasmin and makes the proteolytic enzyme unavailable to mediate clearance of amyloid β fibrils from the neuronal tissue

Among the several enzymes that are involved in Aβ degradation, plasmin plays a critical role in cleaving Aβ and facilitating its clearance [152]. Previous studies have reported reduced plasmin proteolytic degradation and clearance of amyloid precursor protein (APP)/Aβ within AD brains. Correspondingly, increased levels of neuroserpin are associated with decreased tPA activity, resulting in reduced Aβ clearance in AD [39]. Concurrently, AD brains show drastically reduced plasmin/tPA activity when compared to age-matched controls. Hence, it is plausible that neuroserpin upregulation may initially protect the CNS by binding with Aβ, yet inadvertently result in a reduction of plasmin proteolytic propensity to clear neuroserpin-Aβ complexes (Fig. 3a) [39]. However, it is still unclear as to what extent neuroserpin upregulation, neuroserpin–plasmin interaction and consequent inhibition of plasmin may affect normal biochemical and disease processes where plasmin would be deemed beneficial, such as in the clearance and degradation of Aβ (Fig. 3b) [7, 36]. In addition to the above, the disruption of the NGF metabolic cascade due to the inability of plasmin to convert precursor proNGF to mature NGF may contribute to AD pathophysiology [75]. Lee et al. [92] has suggested that increased neuroserpin expression in brain may be the result of uncontrolled neuronal excitation and toxicity [92, 97]. Recent studies have indicated that stimulation of thyroid hormone binding to its receptor thyroid hormone receptor 1-β (THR1β) might be responsible for up-regulation of neuroserpin in AD [148]. Additionally, autophagy impairment has gained considerable attention in recent years in AD and other neurological disorders associated with accumulation of misfolded proteins in the cells [32]. While the endoplasmic reticulum-associated degradation (ERAD) pathway has been mainly implicated for degradation of mutant neuroserpin, autophagy was shown to mediate degradation and turnover of both wild type (WT) and mutant forms of neuroserpin [86]. These observations may provide insights into reasons underlying elevated neuroserpin levels in AD brain tissue throughout the development and progression of the disease.

## Neuroserpin roles in the retina

Early studies identified the presence of neuroserpin as an axonally secreted protein in embryonic chicken retina [145]. Small quantities of the neuroserpin transcript were detected in E14 embryonic chicken retina utilising northern blotting [117]. The expression of neuroserpin was further detected in the ganglion cell and inner nuclear layers of the embryonic retina, with maximum expression prior to and following hatching. This expression was found to be evident at stage 36 of development (as per the chick developmental stages documented by Hamburger and Hamilton), with retinal projections reaching the optic tectum [156]. Strong cellular signals of neuroserpin pointed towards its role in early-stage embryonic synaptogenesis and at this stage of development, retinal projections start developing synaptic connections, which may provide clues on the role of neuroserpin as a modulator for retinal synaptogenesis and remodelling [117].

The mRNA transcripts of neuroserpin are also detectable in both the ganglion cell layer (GCL) and inner nuclear layer (INL) in adult retinas. However, little to barely detectable expression of neuroserpin was evident in the deeper plexiform layers or in the photoreceptors at this point [117]. More recently, wide distribution of neuroserpin expression throughout the retina, with strong expression throughout the GCL and down to the nuclear and plexiform layers of healthy control mice has been reported [55]. Neuroserpin is also abundantly present in the optic nerve head and vitreous under normal physiological conditions [55]. These observations suggest that the serpin might be playing a more broader role in regulation of serine protease activity in the retina [116].

There are reports which implicate the involvement of plasminogen activators in excitotoxicity-induced damage to the retinal neurons in glaucoma conditions [23, 103]. Enhanced plasminogen immunoreactivity has been reported in glaucomatous retinas, particularly within the plexiform layers and surrounding ECM regions [23]. Our group has shown that neuroserpin and plasmin interact under glaucomatous conditions resulting in protease inhibitor complex formation, which was observed to be significantly increased or alternatively stabilised under ocular hypertensive conditions in both human and animal tissues [55]. Retinal immunoprecipitation analysis indicated that this complex is detected as a higher molecular mass band in the human glaucoma samples and in rat models of experimental glaucoma. The expression of the neuroserpin protein however was not altered in the retina, optic nerve head or vitreous of human control and glaucoma samples. Similarly, no intraocular pressure (IOP)-induced changes in neuroserpin and plasmin expression or their localisation within the retina were identified in animal models of glaucoma. In contrast, the plasmin inhibitory activity of neuroserpin was significantly diminished in glaucoma samples from both human retina and in animal models. Consequently, in the absence of an effective proteolytic inhibitory mechanism, human glaucoma retinal samples exhibited enhanced plasmin amidolytic activity, when compared to the controls. Similarly, glaucoma retinal tissues from rat model also showed significantly enhanced plasmin activity. The protein expression analysis of human samples revealed no significant changes for plasmin, tPA or uPA, suggesting that apparent increase in plasmin proteolytic activity, was due to the chronic loss of neuroserpin protease inhibitory activity [55].

Furthermore, studies from our group and others have shown evidence of ECM degradation around the optic nerve head region that coincided with enhanced plasmin activity [54, 68]. This was supported by increased collagen and laminin degradation products identified in the human and rat retinal tissues exposed to glaucoma [55]. Complementary findings have been reported in ischemic stroke mouse models where tPA or plasmin impairment effectively blocked degradation of laminin and protected the neurons [21].

The primary structure of the neuroserpin protein includes 20 Met residues, which are susceptible to oxidation and can become oxidised into methionine sulfoxide. This provides an ancillary anti-oxidant mechanism inherent to the molecule [108]. Application of recombinant neuroserpin to hippocampal neurons in vitro indeed attenuated H2O2 induced oxidative stress effects [22]. Increased methionine sulfoxide immunoreactivity was observed in human glaucoma retinal samples. An increase in methionine sulfoxide reactivity was also evident in the rat glaucomatous retinas. In both cases, methionine sulfoxide staining was predominantly localised to the GCL and INL regions [55]. An important feature of neuroserpin is that it contains a Met residue at its reactive site loop and oxidation may render the molecule inactive, paving way for unregulated plasmin proteolytic actions [108]. Increased methionine sulfoxide reactivity coupled with reduced inhibitory activity, suggests oxidative inactivation of neuroserpin at its reactive site loop Met residues under glaucoma conditions [55].

The propensity of the neuroserpin molecule to undergo oxidative inactivation was also evident from observations in superoxide dismutase 1 (SOD1)-deficient mice. Superoxide dismutase (SOD) mutant mice are characterised by increased oxidative stress; and the neuroserpin molecule demonstrated increased methionine sulfoxide reactivity and loss of inhibitory activity in these animals [55]. This also corresponded with enhanced plasmin proteolytic activity in their retinal tissues. These observations are substantiated by the fact that SOD1-deficient mice display progressive loss of retinal electrophysiology amplitudes, swollen or degenerated mitochondria and thinning of the retinal ganglion cell (RGC) layer, highlighting a potential mechanistic role of neuroserpin inactivation in retinal phenotypes associated with SOD1 loss [63].

Neuroserpin has also been shown to impart functional and retinal laminar structural protection in ischemic models of retinal ganglion cell damage [50]. Endogenous neuroserpin levels increased immediately following ischemic reperfusion injury and a sustained elevation of the protein was observed up to 24 h, particularly within the GCL [50]. Eyes treated with exogenous recombinant neuroserpin showed recovery of electroretinogram (ERG) b-wave amplitudes at seven days post injury, suggesting a neuroprotective role for neuroserpin in the disease model. Further, exogenous neuroserpin was able to significantly attenuate the number of apoptotic TUNEL-positive cells throughout the GCL, INL and outer nuclear layer (ONL) within 24 h of ischemic reperfusion injury, supporting its key role in inner retinal protection [50].

The protective effects of neuroserpin were reinforced by observations in tPA^−/−^ mice, where neuroserpin administration imparted protection against ischemic reperfusion injury. This was evidenced by restoration of the b-wave amplitudes seven days post injury, as well as evidence of reduced TUNEL-staining in neuroserpin-treated mice eyes 24 h post injury [50]. A marked decline in the expression of cleaved caspase-3 and poly-(adenosine diphosphate ribose) polymerase (PARP) was also noted subsequent to neuroserpin treatment. These observations implicate that neuroserpin imparts retinal neuroprotection in both a tPA-dependent and -independent manner, likely due to the inhibition of caspase-3 and caspase-9 cell death signalling pathways [50]. It is important to mention that neuroserpin may accomplish tPA-independent protective effects due to the inhibition of plasmin-induced excitotoxicity and apoptosis, and indeed recent studies have established that neuroserpin can inhibit plasmin activity and that the two proteins interact in the retina [55]. Gu et al. [50] discussed the possibility of neuroserpin being less protective in the absence of tPA, since the effect of neuroserpin was noticed to be less beneficial in tPA^−/−^ mice, albeit non-significantly [50]. Future studies should aim to identify the key cellular signalling pathways affected upon neuroserpin loss, as well as the neuroprotective pathways that are stimulated upon administration of the protein in the retina.

## Neuroserpin polymerization and FENIB

Familial encephalopathy with neuroserpin inclusion bodies (FENIB) is caused by mutations that typically result in opening of the β-sheet-A of neuroserpin molecule. The molecular basis for FENIB manifestation was initially suggested to be based on a loop–sheet model, which proposed that the RCL of one mutant neuroserpin molecule, inserted into the β-sheet-A of another mutant neuroserpin molecule, resulting in the formation and accumulation of loop–sheet polymeric structures within the endoplasmic reticulum (ER) of neuronal cells [30, 107]. However, the formation of serpin polymers is currently hypothesised to be mediated by domain-swapping of the carboxyl-terminal ends of the mutant serpin molecules [26, 40, 169]. Yet despite these developments, the crystal structure of polymerised neuroserpin remains to be established for a better understanding of FENIB neuropathology. The mutant neuroserpin protease inhibitor has been shown to interact poorly with tPA and lead to the rapid formation of polymers [9, 11]. Various FENIB mutations have also previously been linked to the development of phenotypic effects, such as progressive myoclonus epilepsy (neuroserpinosis) [132]. Earlier studies have highlighted the association of unregulated tPA excitotoxicity with the propagation of seizures in wild-type Sprague Dawley rats subjected to intracerebral kainic acid injection [172, 173]. However, a link between the regulation of tPA proteolytic activity by mutant neuroserpin and seizures has yet to be proven in the context of FENIB pathology. Other clinical manifestations of FENIB include progressive cognitive deficits and dementia [14, 30, 132].

The histopathology of FENIB-afflicted individuals was noted by Davis et al. [28, 30] to include mutant neuroserpin inclusion bodies, initially termed Collins bodies, which were distributed throughout the grey matter of the cerebral cortex and to a lesser extent in the subcortical nuclei. These bodies were particularly dispersed amongst the neuropil region and within vacuoles. However, all inclusion bodies were found within neuronal cells and gross examination of these cells showed displaced nuclei and seemingly no cytoplasm [28]. Analysis of both human and transgenic FENIB-mice neuronal tissue also noted that neuroserpin inclusion body formation preceded neurodegeneration and cognitive deficits [29, 44]. Further, brain tissues available from either biopsy or autopsy were analysed and established that the onset and severity of FENIB-associated neurodegeneration and dementia, was directly correlated with the rate of mutant neuroserpin formation and its retention [29, 106]. However, the mechanisms behind its accumulation, or decreased clearance are still poorly understood. FENIB patients exhibit cognitive deficits, such as reduced attention, the loss of oral fluency, concentration, and response regulation function. Magnetic resonance imaging (MRI) studies reveal frontal and frontal subcortical deficits, showing moderate cognitive degeneration, while severe cases demonstrated global cortical atrophy. Furthermore, it is suggested that neuroserpin polymer formation at the neuronal synapse may give rise to chronic localised inflammatory changes, thus participating in the loss of synaptic plasticity in FENIB-associated neurodegeneration [14]

**Fig. 4:**
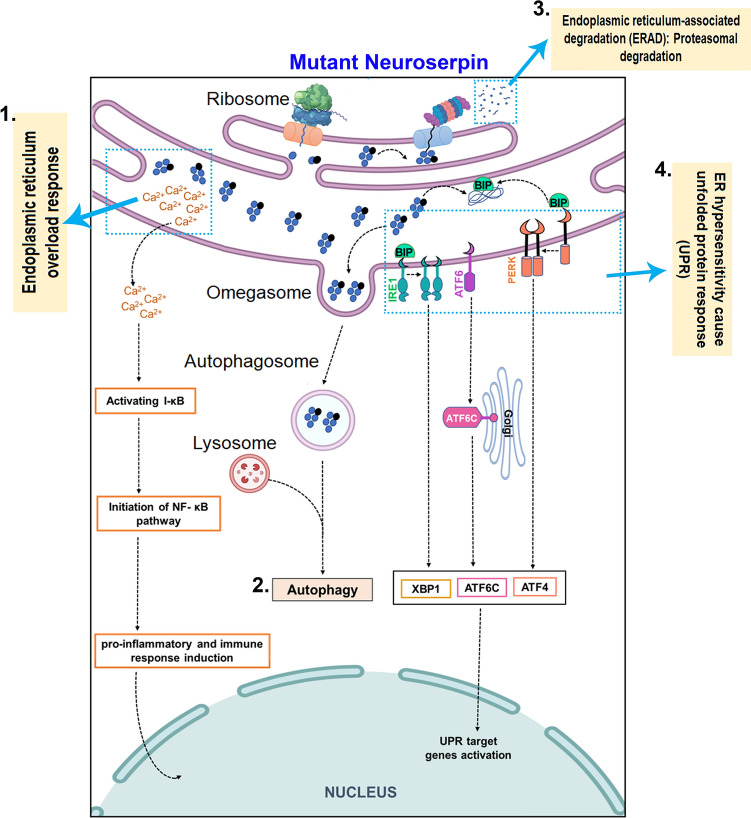
Activation of ER signalling pathways in FENIB. Neuroserpin gene mutations have been associated with inducing endoplasmic reticulum stress response signalling. (1) Mutant neuroserpin polymers stimulate Ca^2+^ efflux from ER leading to I-kB activation and NF-kB mediated pro-inflammatory signalling. (2) Mutant neuroserpin is in part degraded by autophagy pathways through the formation of autophagosomes and their interaction with lysosomes. (3) Endoplasmic reticulum-associated degradation (ERAD) is able to eliminate mutant neuroserpin through proteasomal degradation (4) Mutant neuroserpin accumulation may lead to enhanced ER stress and activation of the unfolded protein response (UPR). These signalling mechanisms eventually lead to either the activation of protein degradation mechanisms or programmed cell death

FENIB pathophysiology is characterised by the accumulation of mutant neuroserpin in the neuronal ER, triggering an ER overload response, with the protein overload potentially activating an altogether different set of stress signalling pathways in diseased conditions, such as NFκB activation, as was observed in the cells in culture [27]. The proteinase inhibitory activity of the serpin and its ability to be secreted is also affected under such conditions [9, 11, 73]. The expression of polymerogenic mutant forms of neuroserpin has been shown to induce the upregulation of antioxidant defence mechanisms in neural cell cultures derived from mice brain, and the disruption of these antioxidant mechanisms was shown to promote pro-apoptotic pathways [52]. The detrimental effects of mutant neuroserpin overexpression were also evident in mice where the formation of inclusion bodies within ER was observed in vivo*,* along with concomitant neuronal loss in various brain regions [44]. The expression of mutant neuroserpin was also recently established to mediate dysregulation of ER morphology in yeast, similar to that observed in mammalian cells expressing mutant neuroserpin [155]. Furthermore, studies in *C. elegans* expressing mutant neuroserpin homolog SRP-2, demonstrated perturbations in heat shock response and unfolded protein response (UPR) signalling pathways (Fig. 4). In these studies, the transient activation of three branches of UPR (inositol-requiring enzyme 1, IRE1; activating transcription factor 6, ATF6; and protein kinase R-like endoplasmic reticulum kinase, PERK) was evident in *C. elegans* and mice models overexpressing mutant forms of neuroserpin at a young age, before the polymers began to accumulate exponentially [137]. The mutant neuroserpin monomers that escape polymerisation are subjected to ER-associated degradation (ERAD) via ligases Hrd1 and gp78, which assist in their ubiquitination, translocation and proteasomal degradation [86, 176]. Overexpression of Hrd1 and gp78 in human embryonic kidney (HEK293) and mouse neuroblastoma (N2a) cells, decreased the level of mutant neuroserpin through the ERAD pathway. In contrast, knockdown of the E3 ubiquitin ligase led to enhancement of G392E aggregates [176]. Further studies have shown that the overexpression of ER-lectin OS-9 (osteosarcoma amplified 9) may lead to the clearance of mutant neuroserpin via ERAD degradation [138]. Ultimately, the significant intracellular accumulation of polymeric neuroserpin elicits an ER overload response, which activates NFκB-mediated cell death signalling (Fig. 4). This NFκB activation is seemingly independent of IRE1, ATF6, and PERK mediated UPR that generally reflects associated ER stress [27]. Similar, NFκB activation independent of UPR effects has previously been observed with polymeric accumulation of another mutant serpin, alpha-1 anti-trypsin [123]. NFκB activation directly correlates with intracellular Ca^2+^ as its depletion is associated with reduced NFκB levels [27]. NF-κB signalling is implicated in regulating neurite growth and the molecule is suggested to play a role in several chronic neurodegenerative conditions [57]. The development of drugs that may restrict propagation of neuroserpin aggregates will be a major therapeutic step forward, as well as enhance our understanding of the biological mechanisms underlying the disease process.

An inverse correlation between cholesterol levels within cell membranes and neuroserpin aggregation, even in the absence of genetic mutation has been observed [49]. Neuroserpin aggregation in the brain was enhanced in response to statin treatment and inhibition of the sterol regulatory binding-element protein (SREBP) [49]. Roussel et al. [133] demonstrated that intracellular cholesterol biosynthetic pathways interact with the neuroserpin mutant G392E polymer, during SREBP-induced activation. A reduced ubiquitination of G392E neuroserpin was observed in vitro, further corroborating a relationship between cholesterol biosynthesis and mutant neuroserpin clearance [133]. N-linked glycosylation of N157 and N321 sites in the molecule has been implicated in attenuating protein polymerisation through their effect on folding and maintaining a functional conformational state [110]. These findings were corroborated in vitro where glycosylated wild-type neuroserpin demonstrated a significantly reduced response to heat-induced polymerisation, whilst non-glycosylated forms were susceptible to aberrant intracellular polymer accumulation and rapid conformational changes when expressed in eukaryotic LEXSY systems and purified [157].

Neuroserpin exhibits significant tertiary structural homology to PAI-1 and given an overlap of interacting proteases between the two molecules, knowledge gained from PAI-1 can potentially help us to design novel neuroserpin ligands for therapeutic applications in FENIB [95]. Embelin is a small molecule antagonist with a molecular weight of 294.39 Da, that interacts with PAI-1; it has also been shown to attenuate neuroserpin polymerisation, without undermining the serpin folding and its tPA proteolytic inhibitory activity [135]. Furthermore, unlike as observed in the case of PAI-1, embelin was able to bind with neuroserpin configurations in native, polymeric, latent, and cleaved states with a particular effect against polymeric and latent conformers that are indicated in FENIB patients. These findings suggest that the interacting motifs of neuroserpin remain unaffected upon polymerisation or C-terminal cleavage. In addition, neuroserpin polymers were also found to disaggregate when incubated with embelin, while native neuroserpin oligomerisation at high temperatures was limited to 2–8 molecules and no formation of latent configuration was evident. Further, while the native neuroserpin/embelin interactions were observed to form oligomers, these remained stable over time and highly soluble. Therefore, these oligomers could hypothetically be secreted and degraded more efficiently by cells. As such, this study proposed that embelin could potentially control neuroserpin polymerisation and find applications in disorders associated with its activation and excessive accumulation in neuronal cells [135]. However, it is acknowledged that while this study demonstrates proof-of-principle for future therapeutic strategies, a greater understanding of these mechanisms and hypothesised cellular degradation pathways in vivo is warranted. Further investigations will enhance our understanding about the roles of neuroserpin in physiological and FENIB disease processes.

## Stroke and neuroprotective effects of neuroserpin

Cerebral ischemic stroke results in the imbalance of energy demand and vascular nutrient supply. Within the cerebral ischemic core, irreversible damage to neurons occurs resulting in tissue necrosis [35]. However, the peripheral regions of the ischemic core, are often the target of many neuroprotective studies, due to the fact that the penumbra may be salvaged via gradual reperfusion from collateral vessels [8]. As such, the current standard of care involves intravenous administration of recombinant tPA to stroke patients, to rapidly reinstate perfusion and increase the therapeutic prospects [1]. This approach employs the intrinsic function of tPA in degrading the fibrin matrix of thrombo-embolic clots to achieve arterial recanalisation [1]. Yet, the caveat is that tPA is subject to a narrow therapeutic window in stroke; and the protein being able to permeate the blood–brain barrier, may promote intracerebral haemorrhage, and induce excitotoxicity damage [12, 79, 174].

The excitotoxic role of tPA in neurodegeneration was initially postulated in studies where both tPA-deficient and wild-type mice were subjected to intra-hippocampal injections of the glutaminergic agonist kainic acid. tPA-deficient mice exhibited increased resistance to neuronal degeneration and seizure, concomitant with attenuated neurotoxic glutamate signalling and microglial activation [151]. The significance of these findings led to the hypothesis that tPA excitotoxicity may lead to extravascular deleterious effects within the brain parenchyma during and following the ischemic stroke [12, 160]. Further, tPA potentiates NMDA receptor-mediated calcium influx, which contributes to excitotoxic neuronal apoptosis [113, 151, 160]. This was conversely exemplified in studies whereby immunotherapeutic prevention of tPA interactions with NMDA receptors was able to reduce cerebral edema and decrease both neuronal apoptosis and microglial activation [43]. Thus, considering the preferential inhibitory activity of neuroserpin on tPA, studies have been investigating the potential neuroprotective role of neuroserpin, as well as the effects of its administration during and following ischemic stroke.

Seminal studies established the rapid increase of neuroserpin immunoreactivity within the ischemic penumbra as early as 6 h after stroke [175]. This was supported by reports that neuroserpin immunoreactivity was completely lost within the ischemic core, and preceded the loss of neuronal marker microtubule-associated protein (MAP)-2 [179]. In a rat model of middle cerebral artery occlusion (MCAO), neuroserpin levels peaked 48 h post ischemia and remained elevated for up to one week [175]. This potentially represents an innate neuroprotective response to ischemia-induced neuronal depolarisation and tPA-dependent excitotoxic cell death [175]. Further, exogenous neuroserpin administration was shown to lead to a significant decrease in the volume of stroke damage and reduced cell death, suggesting increases in neuroserpin levels is protective in ischemic stroke [175]. The intra-cortical administration of neuroserpin was also demonstrated to reduce the extent of laminin degradation induced by microglial activation [175]. A growing body of evidence has since corroborated these findings and exemplified that the role of neuroserpin within the brain parenchyma is indeed associated with neuroprotection, via the attenuation of various excitotoxic and inflammatory factors, mainly mediated by tPA [25]. Transgenic mice overexpressing neuroserpin demonstrated a 30% decrease of cerebral infarct volumes following MCAO induction. Immuno-histochemical analysis and in situ hybridisation of the tissues in this study have revealed diminished microglial activation and reduced levels of plasminogen activators, tPA and uPA [25].

Neuroserpin levels in the brain during ischemic stroke are paralleled by an exacerbated pro-inflammatory pathway response due to microglial activation [47]. The role of microglial activation following ischemic stroke is multifaceted and has been implicated to play both a neurotoxic and neuroprotective role [170]. Microglial activation is instrumental in the phagocytosis of debris, ECM remodelling and the secretion of immunomodulatory cytokines/trophic factors, which are essential for tissue repair following ischemia [170]. Conversely, microglial-induced imbalance of regenerative mechanisms, render neurons susceptible to further neurotoxic injury and cell death [88]. The pro-inflammatory role of microglial activation is also associated with enhanced glutamate, superoxide, nitric oxide, TNFα and matrix metalloproteinase levels [19, 72, 150]. Serpin deficiency was also associated with larger infarct size and worsened neurological outcomes in the mouse model [47].

The detrimental effects of neuroserpin deficiency have also been identified in stroke co-morbidity with diabetes, where diabetic rats following MCAO induction exhibited an increased susceptibility to ischemic injury [94, 161]. Diabetes concomitant to ischemic stroke is associated with evidence of enhanced infarct size, oedema, blood–brain barrier (BBB) dysfunction [80], and is believed to be mediated via sustained exposure to hyperglycaemia induced oxidative stress, Ca^2+^ toxicity, matrix metalloproteinase-9 (MMP-9) activation and the increased expression of the inflammatory marker intracellular adhesion molecule-1 (ICAM-1) [33, 80, 178]. Evidently, other studies noted that decreased neuroserpin levels were inversely correlated with ICAM-1, interleukin 6 (IL-6), MMP-9 and cellular fibronectin (cFn) expression [131]. Moreover, mRNA levels of plasminogen activators tPA, uPA and serpins PAI-1 and neuroserpin were elevated in normal mice following reperfusion. However, neuroserpin levels were significantly reduced in diabetic mice [94]. Thus, it was hypothesised that the exacerbated injury in diabetic mice is linked to reduced fibrinolytic activity, and in later stages aggravated by reduced neuroserpin levels and leakage of tPA into the parenchyma [94]. Accordingly, while neuroserpin potentially displays a neuroprotective effect by limiting tPA-mediated glutaminergic signalling, it has also been implicated in modulating BBB permeability during stroke [131]. The importance of early therapeutic intervention is critical to the survival of neurons not only in ischemic stroke, but also in traumatic brain injury (TBI), which is often linked to haemorrhagic stroke. Interestingly, increased levels of neuroserpin and its enhanced binding with tPA were observed in rat models of acute TBI that were subjected to treatment with progesterone (PROG). These findings suggest a potential role for neuroserpin in mediating the neurotoxic effects of tPA in both ischemic and haemorrhagic stroke [154]. Subsequent studies demonstrated that PROG/allopregnanolone administration may attenuate dysfunction of BBB permeability and reduce infarct size and tPA-mediated inflammation in ischemic stroke animal models [74]. Taken together, the above studies highlight a complex interaction of neuroserpin with tPA proteolytic activity throughout the therapeutic window following stroke.

Cumulative evidence has established that tPA administered one hour after MCAO induction significantly reduced infarct size and increased neurological outcomes [78], in comparison to late tPA-induced thrombolysis which was associated with larger infarct size, BBB leakage and edema within the ischemic core [179]. The intra-cisternal administration of neuroserpin three hours post ischemia significantly reduced this deleterious effect, and widened the tPA therapeutic window, possibly by inhibiting the pleiotropic extravascular effects of tPA within the brain parenchyma [179]. Additionally, the excitotoxicity effects of tPA are associated with ionotropic NMDA receptor perturbations, that lead to Ca^2+^ toxicity and induce superoxide-mediated oxidative stress in neurons [89, 128]. Neuroserpin selectively restricts NMDA receptor mediated intracellular Ca^2+^ influx and its toxic effects, but not α-amino-3-hydroxy-5-methyl-4-isoxazolepropionic acid (AMPA) induced excitotoxicity [89]. Thus, neuroserpin administration may exert neuroprotection due to its inhibitory effects on NMDA-induced toxicity and resultant tissue necrosis (Fig. 2e). Accordingly, neuroserpin-mediated inhibition of NMDA receptors led to increased neuronal survival throughout the cortex and striatum of MCAO-induced mice. Neuroserpin administration also reduced lesion size in mice by attenuating tPA glutaminergic signalling, and intra-neuronal Ca^2+^ spiking. Similar effects were observed in neuronal cultures, which revealed diminished NMDA-mediated Ca^2+^ influx subsequent to neuroserpin treatment [89]. Of interest is that, neuroserpin inhibition of NMDA receptor has also been shown to involve a component that is independent of tPA-mediated effects suggesting that there may be an alternative plasmin regulatory mechanism [165]. This is supported by the observations that plasmin-mediated excitotoxicity was minimised upon either neuroserpin or NMDA receptor antagonist Dizocilpine (MK-801) administration, suggesting a regulatory role of neuroserpin in plasmin-induced excitotoxicity mechanism [165]. The neuroprotective effects of neuroserpin were also evident from tPA knockout mice studies that demonstrated an increased neuroprotection in the absence of tPA, in a sub-lethal injury model of bilateral common carotid artery occlusion [165]. Such injury was shown to be accompanied by an increase in neuroserpin levels in the hippocampal CA1 layer and cerebral cortex, which may induce tolerance to ischemic injury in neuronal cells [165]. The exact dynamics of neuroserpin alterations, region-specific localisation and in vivo concentrations are largely unknown at present, and this remains a challenge to achieve the therapeutic potential of the molecule. Exogenously administered neuroserpin at possibly high concentrations could promote non-specific interactions with other serine proteinases leading to non-discriminate and toxicity effects [92, 165]. However, it is important to highlight that while excitotoxin-induced apoptosis by tPA is plasminogen-dependent [151], tPA may also act independent of the proteolytic enzyme in ischemic-induced cell damage [112]. LRP facilitates trans-endothelial transport across the BBB [31], and this protein is shown to interact with and internalise endogenous tPA [174]. Thus, tPA regulation via LRP is suggested to play a role in the regulation of vascular permeability during stroke [174]. Neuroserpin has been shown to inhibit the effects of tPA-mediated permeabilisation and break down of the BBB, and consequently reduces tPA-induced vasogenic edema [3, 174] in the event of uncontrolled tPA internalisation [174].

## Molecular participation of neuroserpin in cellular proliferation and cancer

A growing body of evidence has implicated a role for neuroserpin in mediating tumour growth, metastasis, and cellular survival pathways. The serpin was demonstrated to be upregulated in poorly differentiated prostate cancer tissue, in comparison to the healthy and well differentiated prostate cancer. Elevated neuroserpin expression was associated with lower survival rates and increased recurrence [65]. Similarly, in lung and breast carcinoma which had metastasised to the brain, neuroserpin expression was found to be elevated approximately three-fold [153]. It is recognised that the plasmin proteolytic axis is involved in multiple metastatic-suppressive mechanisms, particularly through its involvement in the degradation of the axonal pathfinding L1 cell-adhesion molecule (L1CAM). L1CAM is specifically expressed by metastatic cancer cells during their invasion of the brain parenchyma and capillaries. Utilising the reactive stromal signals, plasmin activates Fas ligands (FasL) into a paracrine death signal for proliferating cells. However, in response to increased neuroserpin-mediated protease inhibitory activity, these metastatic-suppressive mechanisms are lost within the brain parenchyma [153]. Alterations in neuroserpin expression were shown to significantly correlate with extent of lung adenocarcinoma metastasis to the brain [126]. In a study comprising 438 non-small cell lung cancer (NSCLC) patient tissues, neuroserpin and adhesion protein L1CAM expression signified correlation with pathological features but did not impinge on the overall survival rates [126, 153]. Rather, neuroserpin overexpression was associated with increased tumour size, tissue necrosis, and pleural cavity invasion. Here, increased L1CAM overexpression was also concomitant with tumour stage, as well as blood and lymph vessel invasion [126]. Previous reports corroborate these findings and suggest that alterations to L1CAM expression was associated with exacerbated tumorigenicity, metastasis, chemoresistance and ultimately a poor prognosis [34, 177]. Additional evidence of neuroserpin involvement is evident in squamous cell carcinoma of the head and neck (SCCHN), where transcription factor p63 was suggested to regulate the neuroserpin expression [51]. p63 participates in cellular proliferation, differentiation and adhesion, and is also conjoined with aggressive and poor prognostic phenotypes [96, 122]; its correlation with neuroserpin may provide a molecular frame-work in regulating cellular migration and metastasis [51].

Cumulative evidence from genetic studies has revealed the existence of a cancer-related gene cluster at the *SERPINI1* locus that codes for neuroserpin, at the 3q26 chromosomal band [18]. This is supported by data emanating from populational studies where variations in neuroserpin genetic region are associated with enhanced risk of adult glioblastoma [125]. Of note, a downregulation of neuroserpin expression has been observed in brain tumour tissue and U-87 MG glioblastoma and H4 neuroglioma cells [18]. Studies have also shown that neuroserpin levels were negatively correlated with tumour progression using northern blot analysis [20]. Later studies investigating the effects of hyper-methylation on cancer-gene silencing have provided evidence that H4 cells treated with de-methylating agents demonstrate increased neuroserpin mRNA levels in a dose-dependent manner [20]. Altogether, this suggests that DNA methylation may be involved in the regulation of neuroserpin transcription, and subsequently aberrant effects of DNA methylation may alter the roles of neuroserpin in cancer metastasis [20].

Neuroserpin mRNA levels have also been shown to increase in childhood brain malignancy atypical teratoid/rhabdoid tumours (AT/RT) [99, 125]. Microarray analysis has revealed a fivefold increase of neuroserpin mRNA in AT/RT when compared to early-stage neuro-ectodermal tumour/medulloblastoma, with which it is frequently misdiagnosed [99]. Recent whole exome sequencing of primary breast tumour and matched brain metastasis patient samples has provided supporting evidence that amongst all serpin family genes, neuroserpin is the most frequently mutated gene implicated in brain metastasis [130], suggesting its role in regulating brain tumorigenesis.

The tumour suppressant roles of neuroserpin have been described in gastric adenocarcinoma and pancreatic cancer [168, 180]. In consideration of that, an inverse correlation between oncogenic microRNA-21 (miR-21) and neuroserpin expression in gastric cancer has been suggested [168]. miR-21 upregulation has been identified in gastric cancer [168], and it is believed to act primarily by decreasing the target RNA levels including that of the neuroserpin expression [53]. Neuroserpin was detected at much lower levels in cancerous stomach tissue, when compared to the controls. Supporting these findings, MKN-28 human gastric carcinoma cells expressing neuroserpin presented a diminutive growth curve, compared to the control cells by inducing vigorous arrest of gap phase 1, during the synthesis phase (G1/S) of the cell cycle [168]. This could suggest that neuroserpin exerts an anti-proliferative effect in these cells. Since cell cycle dysregulation at the gap phase 1 (G1) checkpoint results in the aberrant cell multiplication typical to cancer [168], the modulation of neuroserpin expression may represent a promising mechanism-based strategy to moderate cell proliferation [164].

Neuroserpin was also shown to have an antagonistic effect on pancreatic cancer cell growth, via inhibition of uPA and its receptor (uPAR)-mediated inflammatory response [180]. The anti-inflammatory activity of neuroserpin was initially demonstrated using aortic allograft models for transplant vasculopathy [111]. Neuroserpin administration revealed similar anti-inflammatory effects by decreasing the infiltration of tumour-associated macrophages, leading to the attenuation of pancreatic cancer cell proliferation in xenograft implants [124, 180]. Thus, a key role of macrophage-mediated tumour angiogenesis and metastasis might be constrained by the inhibitory effects of neuroserpin on ECM metalloproteinases, tPA/uPA and plasmin, enzymes which are pivotal to facilitate the immune cell migration and adhesion [180].

Notwithstanding the attenuating effects of neuroserpin on gastric and pancreatic cancers described above, microarray analysis has demonstrated that neuroserpin overexpression is linked with cases of human hepatocellular carcinoma (HCC), where significantly increased neuroserpin expression was observed in advanced stage HCC specimens [77]. Notably, neuroserpin is also elevated in patients with normal serum a-fetoprotein (AFP). While high AFP remains a common biomarker in HCC, normal AFP levels are observed in 30–40% of patients in conjunction with the poor detection of early-stage HCC, which contributes to delayed diagnosis and decreased survival rates in HCC patients [77, 149]. The significance of these findings is that apart from traditional AFP serum biomarker detection, increased neuroserpin expression may serve as an additional early biomarker for HCC. These findings are also corroborated by more recent studies that have identified differential expression of neuroserpin in HCC [143]. Significantly increased neuroserpin levels within the peripheral blood of HCC patients, when compared to cirrhotic and hepatitis C patients has also been reported [134]. Although this particular study could not establish the high specificity and sensitivity of neuroserpin observed in normal level AFP patients as reported by Jia et al. [77],the differences could be attributed to ethnic heterogeneity of the selected cohorts [77, 134].

Studies investigating liver metastasis in colorectal carcinoma have also highlighted a role for neuroserpin in mediating cancer metastasis and cellular adhesion [5]. Here, highly metastatic KM12SM colorectal cancer cells demonstrated significantly increased expression of neuroserpin, when compared to the poorly metastatic parental KM12C cell line [5]. Of note, subsequent neuroserpin gene silencing culminated in a threefold loss of cellular adhesion competency and cell proliferation potential [5]. This may be ascribed to the role of neuroserpin in promoting epithelial-mesenchymal transition (EMT) in vivo, as was demonstrated using orthotopically implanted colorectal cancer models [104]. Metastatic cells are marked by the loss of epithelial cell molecular markers such as E-cadherin and express mesenchymal-like metastatic markers, such as N-cadherin, vimentin and B-catenin [76]. Neuroserpin expression is significantly increased in EMT phenotypic cells when compared to epithelial cells and particularly at the invasive tumour boundaries, when compared to central cancer regions [104]. Other studies have shown that neuroserpin also regulates the cellular adhesion molecule N-cadherin in pheochromocytoma PC12 cells, independently of tPA [90]. This suggests that neuroserpin can promote metastasis independent of its interactions with plasminogen activators [153]. This was supported by the observations that expression of both wild-type neuroserpin as well as its mutant form that lacks tPA inhibitory activity, led to cluster formation phenotype in PC12 cells [90]; whereas the cells that had a reduced neuroserpin expression consistently grew distinctively as single cells. Evidently, the highest levels of N-cadherin are localised to regions of cell–cell contact at the plasma membrane in neuroserpin overexpressed cell lines [90].

Collectively, these studies implicate a key role of neuroserpin in regulating cellular proliferation and metastasis in a cell- and tissue-specific manner. These roles may be mediated via the effects of neuroserpin on plasmin proteolytic axis but also seem to be mediated in part through cellular signalling networks that are independent of this proteolytic pathway. Further investigations will elucidate the crosstalk of neuroserpin with its cellular adhesion interaction partners. This will help understand neuroserpin roles in cancer pathophysiology and potential targeting of the molecule for the development of new anti-proliferative therapies. Finally, neuroserpin may also serve as a candidate biomarker gene in various forms of cancers with applications to improve disease diagnosis and leading to better clinical outcomes.

## Conclusion and future perspectives

In summary, key pathological mechanisms underlying various neurological disorders remain indeterminate, and this has led to limited progress in our ability to therapeutically manage these conditions. Therefore, an in-depth understanding of various aspects of the disease pathology, and novel diagnostic and treatment strategies are needed. Neuroserpin inactivation and protein polymerisation leading to the loss of protein function have been implicated in multiple disorders [30, 47, 55]. Such biochemical aberrations may lead to the suppression of neuroserpin activity below a certain threshold necessary for the protein to impart its protective effects. On the other hand, neuroserpin overexpression as observed in various types of cancer and mutations in neuroserpin, as reported in brain metastasis, are also pathological effects associated with the molecule [99, 130, 153]. The consequences of the mutation have yet to be elucidated, but it can be postulated that the loss of neuroserpin activity, may lead to a gain of biological function in such conditions. Future research should examine neuroserpin augmentation therapy, either in the form of protein infusions or gene therapy to reinstate the protein function in various neurological conditions associated with this protein in the retinal and brain tissues. In recent times, advances in computational biology and drug designing have made it feasible to screen small molecule libraries. The ligands that may bind to serpin hydrophobic pockets and restrict its polymerisation, or molecules that interact with protein reactive site and promote its inhibitory activity will be helpful in drug development [10, 119]. Another potential strategy could be to target the downstream effects of the neuroserpin impairment, rather than the polymerised or oxidatively inactivated protein itself in FENIB, glaucoma, or other disorders associated with the protein. Our ability to achieve selectivity when targeting neuroserpin reactive site loop or other motifs in the molecule will be a major challenge in this direction.

## Data Availability

No data have been generated in the completion of this review article.

## Abbreviations

AD: Alzheimer’s disease
AFP: Serum a-fetoprotein
Akt: Protein kinase B
ALLO: Allopregnanolone
APP: Amyloid precursor protein
AMPA: α-Amino-3-hydroxy-5-methyl-4-isoxazolepropionic acid
Aβ: Amyloid-beta
Aβ40: Amyloid-beta 40
Aβ42: Amyloid-beta 42
AT/RT: Atypical teratoid/rhabdoid tumours
ATF4: Activating transcription factor 4
ATF6: Activating transcription factor 6
ATF6C: Cleaved activating transcription factor 6
AtT20: Cells derived from an anterior pituitary corticotrope tumour
BBB: Blood–brain barrier
Bcl-1: B-cell leukemia line
Bcl-2: B-cell lymphoma 2
BiP: Binding immunoglobulin protein
CA1: Hippocampal cornu ammonis
Cas-3: Caspase-3
cFn: Cellular fibronectin
CNS: Central nervous system
CSF: Cerebrospinal fluid
Cys: Cysteine
Dab1: Disabled-1 protein
DNA: Deoxyribonucleic acid
E3: E3 ubiquitin ligase
ECM: Extracellular matrix
EMT: Epithelial–mesenchymal transition
ER: Endoplasmic reticulum
ERAD: Endoplasmic reticulum-associated degradation
ERG: Electroretinogram
FasL: Fas ligands
FENIB: Familial encephalopathy with neuroserpin inclusion bodies
G1: Gap phase 1
G1/S: Gap phase 1, synthesis phase
GCL: Ganglion cell layer
gp78: Autocrine motility factor receptor
GTPase: Guanosine triphosphate enzyme
HCC: Human hepatocellular carcinoma
HEK293: Human embryonic kidney 293 cells
Hrd1: HMG-CoA reductase degradation protein 1
I-κB: Inhibitor of NF-κB
ICAM1: Inflammatory marker intracellular adhesion molecule-1
IL-6: Interleukin 6
INL: Inner nuclear layer
iPSCs: Induced pluripotent stem cells
IOP: Intra ocular pressure
IRE1: Inositol-requiring enzyme 1
L1CAM: L1 cell-adhesion molecule
LDH: Lactate dehydrogenase
LDL: Low-density lipoprotein
LEXSY: Leishmania expression system
LRP-1: Low-density lipoprotein receptor-related protein 1
MAP2: Microtubule-associated protein 2
MAPK: Mitogen-activated protein kinase
MCAO: Middle cerebral artery occlusion
MCI: Mild cognitive impairment
Met: Methionine
miR-21: MicroRNA-21
MK-801: Dizoclipine
MMP-9: Matrix metalloproteinase-9
MRI: Magnetic resonance imaging
mRNA: Messenger ribonucleic acid
MMSE: Mini-mental state exam
mNGF: Mature neurite growth factor
N2a: Neuro2a mouse neuroblastoma cell line
NFκB: Nuclear factor kappa light chain enhancer of activated B cells
NGF: Nerve growth factor
NMDA: N-Methyl-d-aspartate
NO: Nitric oxide
NSCLC: Non-small cell lung cancer
ONL: Outer nuclear layer
OS-9: Osteosarcoma amplified 9
p63: Transcription factor p63
p65: Transcription factor p65
PAI-1: Plasminogen activator inhibitor-1
PARP: Poly(adenosine diphosphate-ribose) polymerase
PC12: Pheochromocytoma cell line
PD: Parkinson’s disease
PERK: Protein kinase R-like endoplasmic reticulum kinase
PI3K/Akt: Phosphoinositide 3-kinase/protein kinase B
PN-1: Protease nexin-1/glia-derived nexin
PNS: Peripheral nervous system
POAG: Primary open angle glaucoma
proNGF: Precursor nerve growth factor
PROG: Progesterone
Q-PCR: Quantitative polymerase chain reaction
RCL: Reactive centre loop
RGC: Retinal ganglion cell
Rgs2: Regulator of G protein signalling 2
SCCHN: Squamous cell carcinoma of the head and neck
SCI: Subjective cognitive impairment
SERPIN: Serine protease inhibitor
SERPINI1: Serine protease inhibitor, clade I, member 1
SOD1: Superoxide dismutase 1
SREBP: Sterol regulatory binding-element protein
SSRI: Selective serotonin reuptake inhibitor
TBI: Traumatic brain injury
THR1β: Thyroid hormone receptor 1-beta
TNFα: Tumour necrosis factor-alpha
tPA: Tissue plasminogen activator
Trk: Tropomyosin receptor kinase
TrkA: Tropomyosin kinase-A
TUNEL: Terminal deoxynucleotidyl transferase dUTP nick end labelling
uPA: Urokinase plasminogen activator
uPAR: Urokinase plasminogen activator receptor
UPR: Unfolded protein response
VLDL: Very low-density lipoprotein
VLDLR: Very-low-density lipoprotein receptor
XBP1: X-box-binding protein 1

## References

[1] Adibhatla RM, Hatcher JF (2008). Tissue plasminogen activator (tPA) and matrix metalloproteinases in the pathogenesis of stroke-therapeutic strategies. CNS Neurol Disord Drug Targets.

[2] Bacskai BJ, Xia MQ, Strickland DK, Rebeck GW, Hyman BT (2000). The endocytic receptor protein LRP also mediates neuronal calcium signaling via *N*-methyl-d-aspartate receptors. Proc Natl Acad Sci USA.

[3] Baker RN, Cancilla PA, Pollock PS (1971). The movement of exogenous protein in experimental cerebral edema: an electron microscopic study after freeze-injury. J Neuropathol Exp Neurol.

[4] Balasubramanian D, Pearson JF, Kennedy MA (2019). Gene expression effects of lithium and valproic acid in a serotonergic cell line. Physiol Genom.

[5] Barderas R, Mendes M, Torres S, Bartolomé RA, López-Lucendo M, Villar-Vázquez R, Peláez-García A, Fuente E, Bonilla F, Casal JI (2013). In-depth characterization of the secretome of colorectal cancer metastatic cells identifies key proteins in cell adhesion, migration, and invasion. Mol Cell Proteom.

[6] Barker R, Kehoe PG, Love S (2012). Activators and inhibitors of the plasminogen system in Alzheimer's disease. J Cell Mol Med.

[7] Barker R, Love S, Kehoe PG (2010). Plasminogen and plasmin in Alzheimer's disease. Brain Res.

[8] Baron JC (2002). Stroke: imaging and differential diagnosis. J Neural Transm Suppl.

[9] Belorgey D, Crowther DC, Mahadeva R, Lomas DA (2002). Mutant neuroserpin (S49P) that causes familial encephalopathy with neuroserpin inclusion bodies is a poor proteinase inhibitor and readily forms polymers in vitro. J Biol Chem.

[10] Belorgey D, Hagglof P, Onda M, Lomas DA (2010). pH-dependent stability of neuroserpin is mediated by histidines 119 and 138; implications for the control of beta-sheet A and polymerization. Protein Sci.

[11] Belorgey D, Sharp LK, Crowther DC, Onda M, Johansson J, Lomas DA (2004). Neuroserpin Portland (Ser52Arg) is trapped as an inactive intermediate that rapidly forms polymers. Eur J Biochem.

[12] Benchenane K, Lopez-Atalaya JP, Fernandez-Monreal M, Touzani O, Vivien D (2004). Equivocal roles of tissue-type plasminogen activator in stroke-induced injury. Trends Neurosci.

[13] Borges VM, Lee TW, Christie DL, Birch NP (2010). Neuroserpin regulates the density of dendritic protrusions and dendritic spine shape in cultured hippocampal neurons. J Neurosci Res.

[14] Bradshaw CB, Davis RL, Shrimpton AE, Holohan PD, Rea CB, Fieglin D, Kent P, Collins GH (2001). Cognitive deficits associated with a recently reported familial neurodegenerative disease: familial encephalopathy with neuroserpin inclusion bodies. Arch Neurol.

[15] Breton-Provencher V, Drummond GT, Sur M (2021). Locus coeruleus norepinephrine in learned behavior: anatomical modularity and spatiotemporal integration in targets. Front Neural Circuits.

[16] Bruno MA, Cuello AC (2006). Activity-dependent release of precursor nerve growth factor, conversion to mature nerve growth factor, and its degradation by a protease cascade. Proc Natl Acad Sci USA.

[17] Bruses JL (2006). N-cadherin signaling in synapse formation and neuronal physiology. Mol Neurobiol.

[18] Chang W-SW, Chang N-T, Lin S-C, Wu C-W, Wu FY-H (2000). Tissue-specific cancer-related serpin gene cluster at human chromosome band 3q26. Genes Chromosom Cancer.

[19] Chen H, Song YS, Chan PH (2009). Inhibition of NADPH oxidase is neuroprotective after ischemia-reperfusion. J Cereb Blood Flow Metab.

[20] Chen PY, Chang WS, Lai YK, Wu CW (2009). c-Myc regulates the coordinated transcription of brain disease-related PDCD10-SERPINI1 bidirectional gene pair. Mol Cell Neurosci.

[21] Chen Z-L, Strickland S (1997). neuronal death in the hippocampus is promoted by plasmin-catalyzed degradation of laminin. Cell.

[22] Cheng Y, Loh YP, Birch NP (2017). Neuroserpin attenuates H_2_O_2_-induced oxidative stress in hippocampal neurons via AKT and BCL-2 signaling pathways. J Mol Neurosci.

[23] Chintala SK (2016). Tissue and urokinase plasminogen activators instigate the degeneration of retinal ganglion cells in a mouse model of glaucoma. Exp Eye Res.

[24] Chiou A, Hägglöf P, Orte A, Chen AY, Dunne PD, Belorgey D, Karlsson-Li S, Lomas DA, Klenerman D (2009). Probing neuroserpin polymerization and interaction with amyloid-β peptides using single molecule fluorescence. Biophys J.

[25] Cinelli P, Madani R, Tsuzuki N, Vallet P, Arras M, Zhao CN, Osterwalder T, Rulicke T, Sonderegger P (2001). Neuroserpin, a neuroprotective factor in focal ischemic stroke. Mol Cell Neurosci.

[26] D’Acunto E, Fra A, Visentin C, Manno M, Ricagno S, Galliciotti G, Miranda E (2021). Neuroserpin: structure, function, physiology and pathology. Cell Mol Life Sci.

[27] Davies MJ, Miranda E, Roussel BD, Kaufman RJ, Marciniak SJ, Lomas DA (2009). Neuroserpin polymers activate NF-κB by a calcium signaling pathway that is independent of the unfolded protein response. J Biol Chem.

[28] Davis RL, Holohan PD, Shrimpton AE, Tatum AH, Daucher J, Collins GH, Todd R, Bradshaw C, Kent P, Feiglin D, Rosenbaum A, Yerby MS, Shaw C-M, Lacbawan F, Lawrence DA (1999). Familial encephalopathy with neuroserpin inclusion bodies. Am J Pathol.

[29] Davis RL, Shrimpton AE, Carrell RW, Lomas DA, Gerhard L, Baumann B, Lawrence DA, Yepes M, Kim TS, Ghetti B, Piccardo P, Takao M, Lacbawan F, Muenke M, Sifers RN, Bradshaw CB, Kent PF, Collins GH, Larocca D, Holohan PD (2002). Association between conformational mutations in neuroserpin and onset and severity of dementia. Lancet.

[30] Davis RL, Shrimpton AE, Holohan PD, Bradshaw C, Feiglink D, Collins GH, Sonderegger P, Kinter J, Beckers LM, Lacbawan F, Krasnewich D, Muenke M, Lawrence DA, Yerby MS, Shaw C-M, Gooptukk B, Elliottkk PR, Finch JT, Carrell RW, Lomas DA (1999). Familial dementia caused by polymerization of mutant neuroserpin. Nature.

[31] Dehouck B, Fenart L, Dehouck M-P, Pierce A, Gr T, Ro C (1997). A new function for the LDL receptor-transcytosis of LDL across the blood–brain barrier. J Cell Biol.

[32] Di Meco A, Curtis ME, Lauretti E, Praticò D (2020). Autophagy dysfunction in Alzheimer’s disease: mechanistic insights and new therapeutic opportunities. Biol Psychiat.

[33] Ding C, He Q, Li PA (2005). Diabetes increases expression of ICAM after a brief period of cerebral ischemia. J Neuroimmunol.

[34] Doberstein K, Wieland A, Lee SBB, Blaheta RAA, Steffen Wedel HM, Schraml P, Pfeilschifter J, Kristiansen G, Gutwein P (2011). L1-CAM expression in ccRCC correlates with shorter patients survival times and confers chemoresistance in renal cell carcinoma cells. Carcinogenesis.

[35] Durukan A, Tatlisumak T (2007). Acute ischemic stroke: overview of major experimental rodent models, pathophysiology, and therapy of focal cerebral ischemia. Pharmacol Biochem Behav.

[36] Eckman EA, Eckman CB (2005). Aβ-degrading enzymes- modulators of Alzheimer’s disease pathogenesis and targets for therapeutic intervention. Biochem Soc Trans.

[37] Eresheim C, Leeb C, Buchegger P, Nimpf J (2014). Signaling by the extracellular matrix protein Reelin promotes granulosa cell proliferation in the chicken follicle. J Biol Chem.

[38] Fabbro S, Schaller K, Seeds NW (2011). Amyloid-beta levels are significantly reduced and spatial memory defects are rescued in a novel neuroserpin-deficient Alzheimer’s disease transgenic mouse model. J Neurochem.

[39] Fabbro S, Seeds NW (2009). Plasminogen activator activity is inhibited while neuroserpin is up-regulated in the Alzheimer disease brain. J Neurochem.

[40] Faull SV, Elliston ELK, Gooptu B, Jagger AM, Aldobiyan I, Redzej A, Badaoui M, Heyer-Chauhan N, Rashid ST, Reynolds GM, Adams DH, Miranda E, Orlova EV, Irving JA, Lomas DA (2020). The structural basis for Z α(1)-antitrypsin polymerization in the liver. Sci Adv.

[41] Findeis MA (2007). The role of amyloid β peptide 42 in Alzheimer's disease. Pharmacol Ther.

[42] Fredriksson L, Stevenson TK, Su EJ, Ragsdale M, Moore S, Craciun S, Schielke GP, Murphy GG, Lawrence DA (2015). Identification of a neurovascular signaling pathway regulating seizures in mice. Ann Clin Transl Neurol.

[43] Gaberel T, Macrez R, Gauberti M, Montagne A, Hebert M, Petersen KU, Touze E, Agin V, Emery E, Ali C, Vivien D (2013). Immunotherapy blocking the tissue plasminogen activator-dependent activation of *N*-methyl-d-aspartate glutamate receptors improves hemorrhagic stroke outcome. Neuropharmacology.

[44] Galliciotti G, Glatzel M, Kinter J, Kozlov SV, Cinelli P, Rülicke T, Sonderegger P (2007). Accumulation of mutant neuroserpin precedes development of clinical symptoms in familial encephalopathy with neuroserpin inclusion bodies. Am J Pathol.

[45] Gao Z, Godbout R (2013). Reelin-disabled-1 signaling in neuronal migration: splicing takes the stage. Cell Mol Life Sci.

[46] Garnock-Jones KP, McCormack PL (2010). Escitalopram: a review of its use in the management of major depressive disorder in adults. CNS Drugs.

[47] Gelderblom M, Neumann M, Ludewig P, Bernreuther C, Krasemann S, Arunachalam P, Gerloff C, Glatzel M, Magnus T (2013). Deficiency in serine protease inhibitor neuroserpin exacerbates ischemic brain injury by increased postischemic inflammation. PLoS ONE.

[48] Gettins PGW (2002). Serpin Structure, Mechanism, and Function. Chem Rev.

[49] Giampietro C, Lionetti MC, Costantini G, Mutti F, Zapperi S, La Porta CA (2017). Cholesterol impairment contributes to neuroserpin aggregation. Sci Rep.

[50] Gu RP, Fu LL, Jiang CH, Xu YF, Wang X, Yu J (2015). Retina is protected by neuroserpin from ischemic/reperfusion-induced injury independent of tissue-type plasminogen activator. PLoS ONE.

[51] Gu X, Coates PJ, Boldrup L, Nylander K (2008). p63 contributes to cell invasion and migration in squamous cell carcinoma of the head and neck. Cancer Lett.

[52] Guadagno NA, Moriconi C, Licursi V, D'Acunto E, Nisi PS, Carucci N, De Jaco A, Cacci E, Negri R, Lupo G, Miranda E (2017). Neuroserpin polymers cause oxidative stress in a neuronal model of the dementia FENIB. Neurobiol Dis.

[53] Guo H, Ingolia NT, Weissman JS, Bartel DP (2010). Mammalian microRNAs predominantly act to decrease target mRNA levels. Nature.

[54] Guo L, Moss SE, Alexander RA, Ali RR, Fitzke FW, Cordeiro MF (2005). Retinal ganglion cell apoptosis in glaucoma is related to intraocular pressure and IOP-induced effects on extracellular matrix. Invest Ophthalmol Vis Sci.

[55] Gupta V, Mirzaei M, Gupta VB, Chitranshi N, Dheer Y, Vander Wall R, Abbasi M, You Y, Chung R, Graham S (2017). Glaucoma is associated with plasmin proteolytic activation mediated through oxidative inactivation of neuroserpin. Sci Rep.

[56] Gupta VK, You Y, Li JC, Klistorner A, Graham SL (2013). Protective effects of 7,8-dihydroxyflavone on retinal ganglion and RGC-5 cells against excitotoxic and oxidative stress. J Mol Neurosci MN.

[57] Gutierrez H, Davies AM (2011). Regulation of neural process growth, elaboration and structural plasticity by NF-kappaB. Trends Neurosci.

[58] Hadar A, Milanesi E, Squassina A, Niola P, Chillotti C, Pasmanik-Chor M, Yaron O, Martásek P, Rehavi M, Weissglas-Volkov D, Shomron N, Gozes I, Gurwitz D (2016). RGS2 expression predicts amyloid-β sensitivity, MCI and Alzheimer's disease: genome-wide transcriptomic profiling and bioinformatics data mining. Transl Psychiatry.

[59] Hakak Y, Walker JR, Li C, Wong WH, Davis KL, Buxbaum JD, Haroutunian V, Fienberg AA (2001). Genome-wide expression analysis reveals dysregulation of myelination-related genes in chronic schizophrenia. Proc Natl Acad Sci USA.

[60] Han S, Fei F, Sun S, Zhang D, Dong Q, Wang X, Wang L (2021). Increased anxiety was found in serpini1 knockout zebrafish larval. Biochem Biophys Res Commun.

[61] Han W, Dang R, Xu P, Li G, Zhou X, Chen L, Guo Y, Yang M, Chen D, Jiang P (2019). Altered fibrinolytic system in rat models of depression and patients with first-episode depression. Neurobiol Stress.

[62] Hanzel CE, Iulita MF, Eyjolfsdottir H, Hjorth E, Schultzberg M, Eriksdotter M, Cuello AC (2014). Analysis of matrix metallo-proteases and the plasminogen system in mild cognitive impairment and Alzheimer's disease cerebrospinal fluid. J Alzheimers Dis.

[63] Hashizume K, Hirasawa M, Imamura Y, Noda S, Shimizu T, Shinoda K, Kurihara T, Noda K, Ozawa Y, Ishida S, Miyake Y, Shirasawa T, Tsubota K (2008). Retinal dysfunction and progressive retinal cell death in SOD1-deficient mice. Am J Pathol.

[64] Hastings GA, Coleman TA, Haudenschild CC, Stefansson S, Smith EP, Barthlow R, Cherry S, Sandkvist M, Lawrence DA (1997). Neuroserpin, a brain-associated inhibitor of tissue plasminogen activator is localized primarily in neurons. J Biol Chem.

[65] Hasumi H, Ishiguro H, Nakamura M, Sugiura S, Osada Y, Miyoshi Y, Fujinami K, Yao M, Hamada K, Yamada-Okabe H, Kubota Y, Uemura H (2005). Neuroserpin (PI-12) is upregulated in high-grade prostate cancer and is associated with survival. Int J Cancer.

[66] Hayashi H, Campenot RB, Vance DE, Vance JE (2007). Apolipoprotein E-containing lipoproteins protect neurons from apoptosis via a signaling pathway involving low-density lipoprotein receptor-related protein-1. J Neurosci.

[67] Hermann M, Reumann R, Schostak K, Kement D, Gelderblom M, Bernreuther C, Frischknecht R, Schipanski A, Marik S, Krasemann S, Sepulveda-Falla D, Schweizer M, Magnus T, Glatzel M, Galliciotti G (2020). Deficits in developmental neurogenesis and dendritic spine maturation in mice lacking the serine protease inhibitor neuroserpin. Mol Cell Neurosci.

[68] Hernandez MR (2000). The optic nerve head in glaucoma: role of astrocytes in tissue remodeling. Prog Retin Eye Res.

[69] Hill RM, Parmar PK, Coates LC, Mezey E, Pearson JF, Birch NP (2000). Neuroserpin is expressed in the pituitary and adrenal glands and induces the extension of neurite-like processes in AtT-20 cells. Biochem J.

[70] Hu L, Boesten LS, May P, Herz J, Bovenschen N, Huisman MV, Berbee JF, Havekes LM, van Vlijmen BJ, Tamsma JT (2006). Macrophage low-density lipoprotein receptor-related protein deficiency enhances atherosclerosis in ApoE/LDLR double knockout mice. Arterioscler Thromb Vasc Biol.

[71] Huntington JA, Read RJ, Carrell RW (2000). Structure of a serpin-protease complex shows inhibition by deformation. Nature.

[72] Iadecola C, Zhang F, Xu X (1995). Inhibition of inducible nitric oxide synthase ameliorates cerebral ischemic damage. Am Physiol Soc.

[73] Ingwersen T, Linnenberg C, D'Acunto E, Temori S, Paolucci I, Wasilewski D, Mohammadi B, Kirchmair J, Glen RC, Miranda E, Glatzel M, Galliciotti G (2021). G392E neuroserpin causing the dementia FENIB is secreted from cells but is not synaptotoxic. Sci Rep.

[74] Ishrat T, Sayeed I, Atif F, Hua F, Stein DG (2010). Progesterone and allopregnanolone attenuate blood-brain barrier dysfunction following permanent focal ischemia by regulating the expression of matrix metalloproteinases. Exp Neurol.

[75] Iulita MF, Bistue Millon MB, Pentz R, Aguilar LF, Do Carmo S, Allard S, Michalski B, Wilson EN, Ducatenzeiler A, Bruno MA, Fahnestock M, Cuello AC (2017). Differential deregulation of NGF and BDNF neurotrophins in a transgenic rat model of Alzheimer's disease. Neurobiol Dis.

[76] Iwatsuki M, Mimori K, Yokobori T, Ishi H, Beppu T, Nakamori S, Baba H, Mori M (2010). Epithelial-mesenchymal transition in cancer development and its clinical significance. Cancer Sci.

[77] Jia HL, Ye QH, Qin LX, Budhu A, Forgues M, Chen Y, Liu YK, Sun HC, Wang L, Lu HZ, Shen F, Tang ZY, Wang XW (2007). Gene expression profiling reveals potential biomarkers of human hepatocellular carcinoma. Clin Cancer Res.

[78] Jiang Q, Zhang RL, Zhang ZG, Ewing JR, Jiang P, Divine GW, Knight RA, Chopp M (2000). Magnetic resonance imaging indexes of therapeutic efficacy of recombinant tissue plasminogen activator treatment of rat at 1 and 4 hours after embolic stroke. J Cereb Blood Flow Metab.

[79] Jin R, Yang G, Li G (2010). Molecular insights and therapeutic targets for blood-brain barrier disruption in ischemic stroke: critical role of matrix metalloproteinases and tissue-type plasminogen activator. Neurobiol Dis.

[80] Kamada H, Yu F, Nito C, Chan PH (2007). Influence of hyperglycemia on oxidative stress and MMP-9 activation after focal cerebral ischemia: reperfusion in rats-relationship to blood-brain barrier dysfunction. Stroke.

[81] Kelley BJ, Petersen RC (2007). Alzheimer's disease and mild cognitive impairment. Neurol Clin.

[82] Kement D, Reumann R, Schostak K, Voß H, Douceau S, Dottermusch M, Schweizer M, Schlüter H, Vivien D, Glatzel M, Galliciotti G (2021). Neuroserpin is strongly expressed in the developing and adult mouse neocortex but its absence does not perturb cortical lamination and synaptic proteome. Front Neuroanat.

[83] Kerman I, Bernard R, Bunney W, Jones E, Schatzberg A, Myers R, Barchas J, Akil H, Watson S, Thompson R (2012). Evidence for transcriptional factor dysregulation in the dorsal raphe nucleus of patients with major depressive disorder. Front Neurosci.

[84] Kinghorn KJ, Crowther DC, Sharp LK, Nerelius C, Davis RL, Chang HT, Green C, Gubb DC, Johansson J, Lomas DA (2006). Neuroserpin binds Abeta and is a neuroprotective component of amyloid plaques in Alzheimer disease. J Biol Chem.

[85] Koenen KC, Amstadter AB, Ruggiero KJ, Acierno R, Galea S, Kilpatrick DG, Gelernter J (2009). RGS2 and generalized anxiety disorder in an epidemiologic sample of hurricane-exposed adults. Depress Anxiety.

[86] Kroeger H, Miranda E, MacLeod I, Perez J, Crowther DC, Marciniak SJ, Lomas DA (2009). Endoplasmic reticulum-associated degradation (ERAD) and autophagy cooperate to degrade polymerogenic mutant serpins. J Biol Chem.

[87] Krueger SR, Ghisu G-P, Cinelli P, Gschwend TP, Osterwalder T, Wolfer DP, Sonderegger P (1997). Expression of neuroserpin, an inhibitor of tissue plasminogen activator, in the developing and adult nervous system of the mouse. J Neurosci.

[88] Lalancette-Hebert M, Gowing G, Simard A, Weng YC, Kriz J (2007). Selective ablation of proliferating microglial cells exacerbates ischemic injury in the brain. J Neurosci.

[89] Lebeurrier N, Liot G, Lopez-Atalaya JP, Orset C, Fernandez-Monreal M, Sonderegger P, Ali C, Vivien D (2005). The brain-specific tissue-type plasminogen activator inhibitor, neuroserpin, protects neurons against excitotoxicity both in vitro and in vivo. Mol Cell Neurosci.

[90] Lee TW, Coates LC, Birch NP (2008). Neuroserpin regulates N-cadherin-mediated cell adhesion independently of its activity as an inhibitor of tissue plasminogen activator. J Neurosci Res.

[91] Lee TW, Tsang VW, Birch NP (2015). Physiological and pathological roles of tissue plasminogen activator and its inhibitor neuroserpin in the nervous system. Front Cell Neurosci.

[92] Lee TW, Tsang VW, Loef EJ, Birch NP (2017). Physiological and pathological functions of neuroserpin: regulation of cellular responses through multiple mechanisms. Semin Cell Dev Biol.

[93] Leygraf A, Hohoff C, Freitag C, Willis-Owen SA, Krakowitzky P, Fritze J, Franke P, Bandelow B, Fimmers R, Flint J, Deckert J (2006). Rgs 2 gene polymorphisms as modulators of anxiety in humans?. J Neural Transm (Vienna).

[94] Liang W, Chuan-Zhen L, Qiang D, Jian Q, Hui-Min R, Bao-Guo X (2004). Reductions in mRNA of the neuroprotective agent, neuroserpin, after cerebral ischemia/reperfusion in diabetic rats. Brain Res.

[95] Lin Z, Jensen JK, Hong Z, Shi X, Hu L, Andreasen PA, Huang M (2013). Structural insight into inactivation of plasminogen activator inhibitor-1 by a small-molecule antagonist. Chem Biol.

[96] Lo Muzio L, Santarelli A, Caltabiano R, Rubini C, Pieramici T, Trevisiol L, Carinci F, Leonardi R, De Lillo A, Lanzafame S, Bufo P, Piattelli A (2005). p63 overexpression associates with poor prognosis in head and neck squamous cell carcinoma. Hum Pathol.

[97] Lochner JE, Honigman LS, Grant WF, Gessford SK, Hansen AB, Silverman MA, Scalettar BA (2006). Activity-dependent release of tissue plasminogen activator from the dendritic spines of hippocampal neurons revealed by live-cell imaging. J Neurobiol.

[98] Lorenz N, Loef EJ, Verdon DJ, Chen C-JJ, Mansell CJ, Angel CE, Brooks AES, Dunbar PR, Birch NP (2015). Human T cell activation induces synaptic translocation and alters expression of the serine protease inhibitor neuroserpin and its target protease. J Leukocyte Biol.

[99] Ma HI, Kao CL, Lee YY, Chiou GY, Tai LK, Lu KH, Huang CS, Chen YW, Chiou SH, Cheng IC, Wong TT (2010). Differential expression profiling between atypical teratoid:rhabdoid and medulloblastoma tumor in vitro and in vivo using microarray analysis. Childs Nerv Syst.

[100] Ma J, Tong Y, Yu D, Mao M (2012). Tissue plasminogen activator-independent roles of neuroserpin in the central nervous system. Neural Regen Res.

[101] Madani R, Kozlov S, Akhmedov A, Cinelli P, Kinter J, Lipp H-P, Sonderegger P, Wolfer DP (2003). Impaired explorative behavior and neophobia in genetically modified mice lacking or overexpressing the extracellular serine protease inhibitor neuroserpin. Mol Cell Neurosci.

[102] Makarova A, Mikhailenko I, Bugge TH, List K, Lawrence DA, Strickland DK (2003). The low density lipoprotein receptor-related protein modulates protease activity in the brain by mediating the cellular internalization of both neuroserpin and neuroserpin-tissue-type plasminogen activator complexes. J Biol Chem.

[103] Mali RS, Cheng M, Chintala SK (2005). Plasminogen activators promote excitotoxicity-induced retinal damage. FASEB J.

[104] Matsuda Y, Miura K, Yamane J, Shima H, Fujibuchi W, Ishida K, Fujishima F, Ohnuma S, Sasaki H, Nagao M, Tanaka N, Satoh K, Naitoh T, Unno M (2016). SERPINI1 regulates epithelial-mesenchymal transition in an orthotopic implantation model of colorectal cancer. Cancer Sci.

[105] Matthews PR, Harrison PJ (2012). A morphometric, immunohistochemical, and in situ hybridization study of the dorsal raphe nucleus in major depression, bipolar disorder, schizophrenia, and suicide. J Affect Disord.

[106] Miranda E, MacLeod I, Davies MJ, Perez J, Romisch K, Crowther DC, Lomas DA (2008). The intracellular accumulation of polymeric neuroserpin explains the severity of the dementia FENIB. Hum Mol Genet.

[107] Miranda E, Romisch K, Lomas DA (2004). Mutants of neuroserpin that cause dementia accumulate as polymers within the endoplasmic reticulum. J Biol Chem.

[108] Mohsenifar A, Lotfi AS, Ranjbar B, Allameh A, Zaker F, Hasani L, Batool EK, Hasannia S (2007). A study of the oxidation-induced conformational and functional changes in neuroserpin. Iran Biomed J.

[109] Monard D (1988). Cell-derived proteases and protease inhibitors as regulators of neurite outgrowth. Trends Neurosci.

[110] Moriconi C, Ordonez A, Lupo G, Gooptu B, Irving JA, Noto R, Martorana V, Manno M, Timpano V, Guadagno NA, Dalton L, Marciniak SJ, Lomas DA, Miranda E (2015). Interactions between N-linked glycosylation and polymerisation of neuroserpin within the endoplasmic reticulum. FEBS J.

[111] Munuswamy-Ramanujam G, Dai E, Liu L, Shnabel M, Sun YM, Bartee M, Lomas DA, Lucas AR (2010). Neuroserpin, a thrombolytic serine protease inhibitor (serpin), blocks transplant vasculopathy with associated modification of T-helper cell subsets. Thromb Haemost.

[112] Nagai N, De Mol M, Lijnen HR, Carmeliet P, Collen D (1999). Role of plasminogen system components in focal cerebral ischemic infarction: a gene targeting and gene transfer study in mice. Circulation.

[113] Nicole O, Docagne F, Ali C, Margaill I, Carmeliet P, Mackenzie ET, Vivien D, Buisson A (2001). The proteolytic activity of tissue-plasminogen activator enhances NMDA receptor-mediated signaling. Nat Med.

[114] Nielsen HM, Minthon L, Londos E, Blennow K, Miranda E, Perez J, Crowther DC, Lomas DA, Janciauskiene SM (2007). Plasma and CSF serpins in Alzheimer disease and dementia with Lewy bodies. Neurology.

[115] Olson ST, Gettins PG (2011). Regulation of proteases by protein inhibitors of the serpin superfamily. Prog Mol Biol Transl Sci.

[116] Osterwalder T, Cinelli P, Baici A, Pennella A, Krueger SR, Schrimpf SP, Meins M, Sonderegger P (1998). The axonally secreted serine proteinase inhibitor, neuroserpin, inhibits plasminogen activators and plasmin but not thrombin. J Biol Chem.

[117] Osterwalder T, Contartese J, Stoeckli ET, Kuhn TB, Sonderegger P (1996). Neuroserpin, an axonally secreted serine protease inhibitor. EMBO J.

[118] Parathath SR, Parathath S, Tsirka SE (2006). Nitric oxide mediates neurodegeneration and breakdown of the blood-brain barrier in tPA-dependent excitotoxic injury in mice. J Cell Sci.

[119] Parfrey H, Dafforn TR, Belorgey D, Lomas DA, Mahadeva R (2004). Inhibiting polymerization: new therapeutic strategies for Z alpha1-antitrypsin-related emphysema. Am J Respir Cell Mol Biol.

[120] Park L, Gallo EF, Anrather J, Wang G, Norris EH, Paul J, Strickland S, Iadecola C (2008). Key role of tissue plasminogen activator in neurovascular coupling. Proc Natl Acad Sci USA.

[121] Parmar PK, Coates LC, Pearson JF, Hill RM, Birch NP (2002). Neuroserpin regulates neurite outgrowth in nerve growth factor-treated PC12 cells. J Neurochem.

[122] Perez CA, Pietenpol JA (2007). Transcriptional programs regulated by p63 in normal epithelium and tumors. Cell Cycle.

[123] Perlmutter DH (2016). alpha1-antitrypsin deficiency: a misfolded secretory protein variant with unique effects on the endoplasmic reticulum. Endoplasmic Reticulum Stress Dis.

[124] Qian BZ, Pollard JW (2010). Macrophage diversity enhances tumor progression and metastasis. Cell.

[125] Rajaraman P, Brenner AV, Butler MA, Wang SS, Pfeiffer RM, Ruder AM, Linet MS, Yeager M, Wang Z, Orr N, Fine HA, Kwon D, Thomas G, Rothman N, Inskip PD, Chanock SJ (2009). Common variation in genes related to innate immunity and risk of adult glioma. Cancer Epidemiol Biomark Prev.

[126] Ramnefjell M, Aamelfot C, Helgeland L, Akslen LA (2017). Low expression of SerpinB2 is associated with reduced survival in lung adenocarcinomas. Oncotarget.

[127] Reumann R, Vierk R, Zhou L, Gries F, Kraus V, Mienert J, Romswinkel E, Morellini F, Ferrer I, Nicolini C, Fahnestock M, Rune G, Glatzel M, Galliciotti G (2017). The serine protease inhibitor neuroserpin is required for normal synaptic plasticity and regulates learning and social behavior. Learn Mem.

[128] Reyes RC, Brennan AM, Shen Y, Baldwin Y, Swanson RA (2012). Activation of neuronal NMDA receptors induces superoxide-mediated oxidative stress in neighboring neurons and astrocytes. J Neurosci.

[129] Ricagno S, Caccia S, Sorrentino G, Antonini G, Bolognesi M (2009). Human neuroserpin: structure and time-dependent inhibition. J Mol Biol.

[130] Richichi C, Fornasari L, Melloni GEM, Brescia P, Patanè M, Del Bene M, Mustafa DAM, Kros JM, Pollo B, Pruneri G, Sciandivasci A, Munzone E, DiMeco F, Pelicci PG, Riva L, Pelicci G (2017). Mutations targeting the coagulation pathway are enriched in brain metastases. Sci Rep.

[131] Rodríguez-González R, Sobrino T, Rodríguez-Yáñez M, Millán M, Brea D, Miranda E, Moldes O, Pérez J, Lomas DA, Leira R, Dávalos A, Castillo J (2011). Association between neuroserpin and molecular markers of brain damage in patients with acute ischemic stroke. J Transl Med.

[132] Roussel BD, Lomas DA, Crowther DC (2016). Progressive myoclonus epilepsy associated with neuroserpin inclusion bodies (neuroserpinosis). Epileptic Disord.

[133] Roussel BD, Newton TM, Malzer E, Simecek N, Haq I, Thomas SE, Burr ML, Lehner PJ, Crowther DC, Marciniak SJ, Lomas DA (2013). Sterol metabolism regulates neuroserpin polymer degradation in the absence of the unfolded protein response in the dementia FENIB. Hum Mol Genet.

[134] Saad Y, El-Serafy M, Eldin MS, Abdellatif Z, Khatab H, Elbaz T, Elgarem H (2013). New genetic markers for diagnosis of hepatitis C related hepatocellular carcinoma in Egyptian patients. J Gastrointestin Liver Dis.

[135] Saga G, Sessa F, Barbiroli A, Santambrogio C, Russo R, Sala M, Raccosta S, Martorana V, Caccia S, Noto R, Moriconi C, Miranda E, Grandori R, Manno M, Bolognesi M, Ricagno S (2016). Embelin binds to human neuroserpin and impairs its polymerisation. Sci Rep.

[136] Santarelli L, Saxe M, Gross C, Surget A, Battaglia F, Dulawa S, Weisstaub N, Lee J, Duman R, Arancio O, Belzung C, Hen R (2003). Requirement of hippocampal neurogenesis for the behavioral effects of antidepressants. Science.

[137] Schipanski A, Lange S, Segref A, Gutschmidt A, Lomas DA, Miranda E, Schweizer M, Hoppe T, Glatzel M (2013). A novel interaction between aging and ER overload in a protein conformational dementia. Genetics.

[138] Schipanski A, Oberhauser F, Neumann M, Lange S, Szalay B, Krasemann S, van Leeuwen FW, Galliciotti G, Glatzel M (2014). The lectin OS-9 delivers mutant neuroserpin to endoplasmic reticulum associated degradation in familial encephalopathy with neuroserpin inclusion bodies. Neurobiol Aging.

[139] Schrimpf SP, Bleker AJ, Brecevic L, Kozlov SV, Berger P, Osterwaler T, Krueger SR, Schinzel A, Sonderegger P (1997). Human neuroserpin (PI12)-cDNA cloning and chromosomal localization to 3q26. Genomics.

[140] Shi Y, Mantuano E, Inoue G, Campana WM, Gonias SL (2009). Ligand binding to LRP1 transactivates Trk receptors by a Src family kinase-dependent pathway. Sci Signal.

[141] Silverman GA, Bird PI, Carrell RW, Church FC, Coughlin PB, Gettins PGW, Irving JA, Lomas DA, Luke CJ, Moyer RW, Pemberton PA, Remold-O'Donnell E, Salvesen GS, Travis J, Whisstock JC (2001). The serpins are an expanding superfamily of structurally similar but functionally diverse proteins. J Biol Chem.

[142] Smoller JW, Paulus MP, Fagerness JA, Purcell S, Yamaki LH, Hirshfeld-Becker D, Biederman J, Rosenbaum JF, Gelernter J, Stein MB (2008). Influence of RGS2 on anxiety-related temperament, personality, and brain function. Arch Gen Psychiatry.

[143] Spano D, Russo R (2010). Galectin-1 and its involvement in hepatocellular carcinoma aggressiveness. Mol Med.

[144] Stein PE, Carrell RW (1995). What do dysfunctional serpins tell us about molecular mobility and disease?. Nat Struct Mol Biol.

[145] Stoeckli ET, Lemkin PF, Kuhn TB, Ruegg MA, Heller M, Songeregger P (1989). Identification of proteins secreted from axons of embryonic dorsal-root-ganglia neurons. Eur J Biochem.

[146] Strickland DK, Muratoglu SC, Antalis TM (2011). Serpin-enzyme receptors LDL receptor-related protein 1. Methods Enzymol.

[147] Su EJ, Fredriksson L, Geyer M, Folestad E, Cale J, Andrae J, Gao Y, Pietras K, Mann K, Yepes M, Strickland DK, Betsholtz C, Eriksson U, Lawrence DA (2008). Activation of PDGF-CC by tissue plasminogen activator impairs blood-brain barrier integrity during ischemic stroke. Nat Med.

[148] Subhadra B, Schaller K, Seeds NW (2013). Neuroserpin up-regulation in the Alzheimer's disease brain is associated with elevated thyroid hormone receptor-β1 and HuD expression. Neurochem Int.

[149] Taketa K (1990). a-Fetoprotein—reevaluation in hepatology. Hepatology.

[150] Takeuchi H, Jin S, Wang J, Zhang G, Kawanokuchi J, Kuno R, Sonobe Y, Mizuno T, Suzumura A (2006). Tumor necrosis factor-alpha induces neurotoxicity via glutamate release from hemichannels of activated microglia in an autocrine manner. J Biol Chem.

[151] Tsirka SE, Gualandris A, Amaral DG, Strickland S (1995). Excitotoxin-induced neuronal degeneration and seizure are mediated by tissue plasminogen activator. Nature.

[152] Tucker HM, Kihiko M, Caldwell JN, Wright S, Kawarabayashi T, Price D, Walker D, Scheff S, McGillis JP, Rydel RE, Estus S (2000). The plasmin system is induced by and degrades amyloid-beta aggregates. J Neurosci.

[153] Valiente M, Obenauf AC, Jin X, Chen Q, Zhang XH, Lee DJ, Chaft JE, Kris MG, Huse JT, Brogi E, Massague J (2014). Serpins promote cancer cell survival and vascular co-option in brain metastasis. Cell.

[154] VanLandingham JW, Cekic M, Cutler SM, Hoffman SW, Washington ER, Johnson SJ, Miller D, Stein DG (2008). Progesterone and its metabolite allopregnanolone differentially regulate hemostatic proteins after traumatic brain injury. J Cereb Blood Flow Metab.

[155] Vapore V, Mazzaglia C, Sibilia D, Del Vecchio M, Fruhmann G, Valenti M, Miranda E, Rinaldi T, Winderickx J, Mazzoni C (2021). Neuroserpin inclusion bodies in a FENIB yeast model. Microorganisms.

[156] Hamburger V, Hamilton HL (1951). A series of normal stages in the development of the chick embryo. J Morphol.

[157] Visentin C, Broggini L, Sala BM, Russo R, Barbiroli A, Santambrogio C, Nonnis S, Dubnovitsky A, Bolognesi M, Miranda E, Achour A, Ricagno S (2020). Glycosylation tunes neuroserpin physiological and pathological properties. Int J Mol Sci.

[158] Wang L, Zhang Y, Asakawa T, Li W, Han S, Li Q, Xiao B, Namba H, Lu C, Dong Q (2015). Neuroprotective effect of neuroserpin in oxygen-glucose deprivation- and reoxygenation-treated rat astrocytes in vitro. PLoS ONE.

[159] Wang Y, Luo W, Reiser G (2008). Trypsin and trypsin-like proteases in the brain: proteolysis and cellular functions. Cell Mol Life Sci.

[160] Wang YF, Tsirka SE, Strickland S, Stieg PE, Soriano SG, Lipton SA (1998). Tissue plasminogen activator (tPA) increases neuronal damage after focal cerebral ischemia in wild-type and tPA-deficient mice. Nat Med.

[161] Wanga G-X, Lia G-R, Wanga Y-D, Yangb T-S, Ouyangb Y-B (2001). Characterization of neuronal cell death in normal and diabetic rats following exprimental focal cerebral ischemia. Life Sci.

[162] Weinberger DR (1987). Implications of normal brain development for the pathogenesis of schizophrenia. Arch Gen Psychiatry.

[163] Wen Z, Nguyen HN, Guo Z, Lalli MA, Wang X, Su Y, Kim NS, Yoon KJ, Shin J, Zhang C, Makri G, Nauen D, Yu H, Guzman E, Chiang CH, Yoritomo N, Kaibuchi K, Zou J, Christian KM, Cheng L, Ross CA, Margolis RL, Chen G, Kosik KS, Song H, Ming GL (2014). Synaptic dysregulation in a human iPS cell model of mental disorders. Nature.

[164] Williams GH, Stoeber K (2012). The cell cycle and cancer. J Pathol.

[165] Wu J, Echeverry R, Guzman J, Yepes M (2010). Neuroserpin protects neurons from ischemia-induced plasmin-mediated cell death independently of tissue-type plasminogen activator inhibition. Am J Pathol.

[166] Yalcin B, Willis-Owen SA, Fullerton J, Meesaq A, Deacon RM, Rawlins JN, Copley RR, Morris AP, Flint J, Mott R (2004). Genetic dissection of a behavioral quantitative trait locus shows that Rgs2 modulates anxiety in mice. Nat Genet.

[167] Yamada M, Takahashi K, Ukai W, Hashimoto E, Saito T, Yamada M (2010). Neuroserpin is expressed in early stage of neurogenesis in adult rat hippocampus. NeuroReport.

[168] Yamanaka S, Olaru AV, An F, Luvsanjav D, Jin Z, Agarwal R, Tomuleasa C, Popescu I, Alexandrescu S, Dima S, Chivu-Economescu M, Montgomery EA, Torbenson M, Meltzer SJ, Selaru FM (2012). MicroRNA-21 inhibits Serpini1, a gene with novel tumour suppressive effects in gastric cancer. Dig Liver Dis.

[169] Yamasaki M, Sendall TJ, Pearce MC, Whisstock JC, Huntington JA (2011). Molecular basis of α1-antitrypsin deficiency revealed by the structure of a domain-swapped trimer. EMBO Rep.

[170] Yenari MA, Kauppinen TM, Swanson RA (2010). Microglial activation in stroke-therapeutic targets. J Am Soc Exp NeuroTher.

[171] Yepes M, Lawrence DA (2004). Neuroserpin: a selective inhibitor of tissue-type plasminogen activator in the central nervous system. Thromb Haemost.

[172] Yepes M, Lawrence DA (2004). Tissue-type plasminogen activator and neuroserpin: a well-balanced act in the nervous system?. Trends Cardiovasc Med.

[173] Yepes M, Sandkvist M, Colman TA, Moore E, Wu J-Y, Mitola D, Bugger TH, Lawrence DA (2002). Regulation of seizure spreading by neuroserpin and tissue-type plasminogen activator is plasminogen-independent. J Clin Invest.

[174] Yepes M, Sandkvist M, Moore EG, Bugge TH, Strickland DK, Lawrence DA (2003). Tissue-type plasminogen activator induces opening of the blood-brain barrier via the LDL receptor-related protein. J Clin Investig.

[175] Yepes M, Sandkvist M, Wong MKK, Coleman TA, Smith E, Cohan SL, Lawrence DA (2000). Neuroserpin reduces cerebral infarct volume and protects neurons from ischemia-induced apoptosis. Blood.

[176] Ying Z, Wang H, Fan H, Wang G (2011). The endoplasmic reticulum (ER)-associated degradation system regulates aggregation and degradation of mutant neuroserpin. J Biol Chem.

[177] Zeimet AG, Reimer D, Huszar M, Winterhoff B, Puistola U, Azim SA, Müller-Holzner E, An B-A, LoCv K, Petru E, Jahn S, Geels YR, Massuger LR, Fdr A, Polterauer S, Lappi-Blanco E, Bulten J, Meuter A, Tanouye S, Oppeit P, Stroh-Weigert M, Reinthaller A, Mariani A, Hackl W, Netzer M, Schirmer U, Vergote I, Altevogt P, Marth C, Fogel M (2013). L1CAM in early-stage type I endometrial cancer—results of a large multicenter evaluation. J Natl Cancer Inst.

[178] Zhang R-L, Lu C-Z, Ren H-M, Xiao B-G (2003). Metabolic changes of arachidonic acid after cerebral ischemia-reperfusion in diabetic rats. Exp Neurol.

[179] Zhang Z, Zhang L, Yepes M, Jiang Q, Li Q, Arniego P, Coleman TA, Lawrence DA, Chopp M (2002). Adjuvant treatment with neuroserpin increases the therapeutic window for tissue-type plasminogen activator administration in a rat model of embolic stroke. Circulation.

[180] Zheng D, Chen H, Bartee MY, Williams J, Davids JA, Lomas DA, McFadden G, Lucas AR (2013). Myxomaviral anti-inflammatory serpin reduces myeloid-derived suppressor cells and human pancreatic cancer cell growth in mice. J Cancer Sci Ther.

